# Selective Personality-Targeted Intervention and the Escalation of Substance Use During Adolescence: A Secondary Analysis of A Cluster-Randomized Clinical Trial

**DOI:** 10.1001/jamanetworkopen.2025.50176

**Published:** 2025-12-18

**Authors:** Samantha J. Lynch, Sherry H. Stewart, Patricia Conrod

**Affiliations:** 1 Department of Psychiatry and Addiction, Faculty of Medicine, University of Montreal, Montreal, Canada; 2 Azrieli Research Center, CHU Ste Justine Mother-Child University Hospital, Montreal, Canada; 3 Department of Psychiatry, Faculty of Medicine, Dalhousie University, Halifax, Canada; 4 Department of Psychology and Neuroscience, Faculty of Science, Dalhousie University, Halifax, Canada; 5 Department of Community Health and Epidemiology, Faculty of Medicine; Dalhousie University, Halifax, Canada

## Abstract

**Importance:**

Substance use is a leading cause of burden of disease worldwide. Selective prevention programs can help reduce the development of problematic substance use and disorders.

**Objective:**

To examine effects of a selective, personality-targeted substance use prevention program on alcohol, cannabis, tobacco, nonmedical opioid, and illicit polysubstance use among adolescents with elevated levels of personality traits associated with frequent and risky substance use.

**Design, Setting, and Participants:**

This prespecified secondary analysis of a cluster-randomized clinical trial assessed the effectiveness of a personality-targeted cognitive-behavioral intervention (PreVenture) in reducing substance use during adolescence. Participants included grade 7 students attending 31 secondary schools in the Greater Montreal Area, Canada (2012-2013 school year), who screened as having elevated levels of anxiety sensitivity, hopelessness, impulsivity, or sensation seeking. Schools were randomized to either the intervention (15 schools) or the control group (16 schools). Data were analyzed in January to March 2025.

**Interventions:**

Schools delivered a 2-session, personality-targeted group-based cognitive-behavioral intervention designed to help students recognize, challenge, and manage personality-specific emotions, behaviors, and cognitions associated with substance use in adolescence.

**Main Outcomes and Measures:**

Self-reported frequency of alcohol, cannabis, smoking tobacco, nonmedical use of opioids, and illicit polysubstance use were measured by the Detection of Alcohol and Drug Problems in Adolescents.

**Results:**

A total of 3861 students from 31 schools were screened; 1669 students (847 [50.7%] female; mean [SD] age, 12.83 [0.47] years) were at elevated risk of substance use and included in the intention-to-treat sample. The 31 schools were randomized, with 16 schools with 964 students in the control group and 15 schools with 705 students in the intervention group. Multilevel bayesian mixed-effects models indicated that students in the intervention group had slower increases in the frequency of alcohol (odds ratio [OR], 0.92; 95% credible interval [CrI], 0.85-1.00), cannabis (OR, 0.75; 95% CrI, 0.66-0.86), tobacco smoking (OR, 0.79; 95% CrI, 0.70-0.96), and illicit polysubstance use (OR, 0.56; 95% CrI, 0.35-0.89) over 4 years. Effects did not differ by sex.

**Conclusions and Relevance:**

This secondary analysis of a cluster-randomized clinical trial found that personality-targeted interventions protected against the escalation in substance use during adolescence, with similar effects for males and females.

**Trial Registration:**

ClinicalTrials.gov Identifier: NCT01655615.

## Key Points

**Question:**

What are the effects of a selective personality-targeted intervention on alcohol, cannabis, tobacco, nonmedical use of opioids, and illicit polysubstance use among adolescents?

**Findings:**

This secondary analysis of 1669 students in a cluster-randomized clinical trial found that the intervention was effective in reducing frequency of substance use and polysubstance use in adolescents.

**Meaning:**

These findings suggest that selective personality-targeted prevention attenuates the escalation of substance use among adolescents reporting early risk factors for problematic substance use.

## Teaser

This secondary analysis of cluster-randomized clinical trial examines effects of a selective, personality-targeted substance use prevention program on alcohol, cannabis, tobacco, nonmedical opioid, and illicit polysubstance use among adolescents with elevated levels of personality traits associated with frequent and risky substance use.

## Introduction

Adolescent substance use is a global public health issue with substantial individual and societal impacts,^1,2,3^ including long-lasting effects on brain development, physical and mental health problems, criminal justice system involvement, and poor academic achievement.^4,5,6^ Early initiation of substance use and use of multiple substances increase the risk of developing a substance use disorder.^6,7^ Despite declining substance use rates among young people,^8,9^ prevalence of substance use disorders^5^ and related harms have increased.^10,11,12,13^ This discrepancy highlights the need for targeted, evidence-based prevention initiatives.

PreVenture is a personality-targeted intervention designed to reduce substance use and related risk behaviors by addressing traits associated with risky substance use and mental health concerns, including sensation seeking, impulsivity, hopelessness, and anxiety sensitivity.^14,15^ The program uses brief, tailored cognitive-behavioral strategies to promote adaptive coping and challenge risky substance use motives, particularly to cope with personality-related vulnerabilities, such as using to get high or to cope with anxiety or depression symptoms or impulsive substance use. This intervention has demonstrated efficacy in delaying substance use initiation and reducing problematic use of alcohol, tobacco, cannabis, and illicit substance use across multiple trials, particularly in the United Kingdom and Australia.^16,17,18,19,20,21,22,23^

Given concurrent changes in North American drug policy (eg, legalization of cannabis) and increasing drug-related harms, it is important to demonstrate the program’s continued efficacy in reducing adolescent substance use in a more precarious substance use landscape. Primary outcomes of the Canadian CoVenture trial indicated that the PreVenture intervention reduced odds of developing a substance use disorder up to 4 years after the intervention.^24^ This study reports secondary substance use outcomes from CoVenture to examine impacts of a personality-targeted selective prevention program on frequency and escalation of risky substance use in Canada during a shift toward more liberal cannabis policies and increasing nonmedical prescription drug use among adolescents.^25,26^ Furthermore, this study expands prior research by directly examining effects on cannabis use, for which results have tended to be mixed or marginal in the past.^27,28,29,30^ The CoVenture trial updated intervention materials to reflect the high rates of cannabis use among young people in Canada.^31,32,33^ While previous versions of the program referred to alcohol exclusively, the program was adapted for the current context to include opportunities to explore how these traits are also implicated in cannabis use and the potential risks of using cannabis to manage these traits.

Recent research has reported associations of the personality traits targeted in the our program with nonmedical and medical prescription drug use,^34^ yet the effects of this intervention on nonmedical prescription opioid use have not previously been examined. Nonmedical prescription drug use and opioid-related harms have increased substantially in Canada.^33,35,36^ A new conceptualization of the North American drug crisis points to the role of polysubstance use in opioid-related morbidity and mortality.^37,38^ Among adolescents, early patterns of polysubstance use are associated with heightened risk of substance use disorders, overdose, and opioid-related harms.^38,39,40^ A recent systematic review^41^ highlighted the lack of evidence-based prevention for opioid use, underscoring the urgent need for evidence-based interventions for nonmedical use of opioids and polysubstance use.

Finally, recent epidemiological research indicates a significant shift in adolescent substance use patterns across sexes. Historically, males reported higher rates of alcohol, cannabis, and other substance use and were more often diagnosed with substance use disorders.^42,43,44,45,46^ However, recent evidence indicates that females are increasingly using substances at rates comparable to, or exceeding, those of males (ie, convergence).^47,48,49,50,51^ Recent declines in youth substance use have occurred more rapidly among males,^9^ and some evidence suggests that females may transition to high-frequency use more rapidly (ie, telescoping).^52^ Furthermore, nonmedical use of prescription drugs is higher among adolescent females in North America.^53,54^ These trends highlight the need for prevention efforts that are responsive to changing substance use patterns across the sexes. Although personality-targeted interventions have shown promise in reducing substance use, little is known about whether the impact differs by sex.

This study evaluated the impact of PreVenture on frequency of substance use among adolescents in Canada, including alcohol, cannabis, tobacco smoking, and nonmedical opioid use. We also examined program effects on illicit polysubstance use, a key contributor to overdose risk and opioid-related harms. Additionally, we explored potential sex differences in the effectiveness of the intervention. By evaluating the intervention’s impact on substance use patterns and escalation during adolescence, we aim to inform evidence-based approaches to the prevention of adolescent substance use and related harms in the context of a drug overdose epidemic.

## Methods

### Study Design and Participants

**Figure 1.**
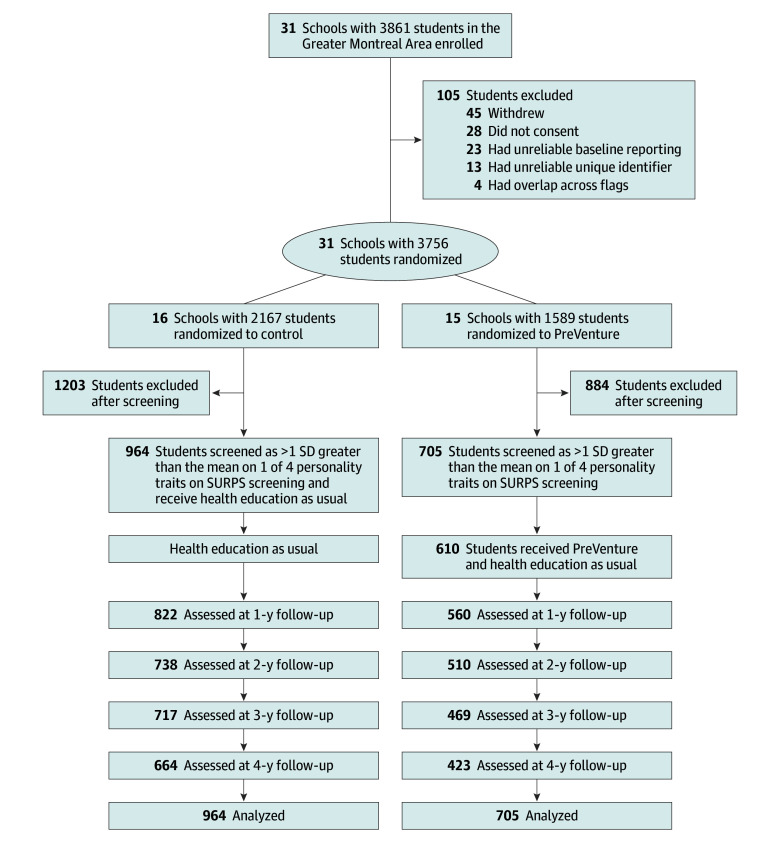
Participant Flow Throughout the CoVenture Trial in the Intervention and Control Groups SURPS indicates Substance Use Risk Profile Scale.

This was a prespecified secondary analysis of CoVenture, a cluster-randomized clinical trial (RCT) conducted between 2012 and 2017. Ethical approval was obtained from the CHU Sainte-Justine Hospital research ethics board. The study followed the Consolidated Standards of Reporting Trials (CONSORT) reporting guideline. A detailed trial protocol has been published elsewhere^31^ and is available in Supplement 1. Eligible participants were grade 7 students (age 12-13 years) from 31 secondary schools in the Greater Montreal, Canada, Area. All students provided active written consent. In addition, 9 schools required parental written consent due to school board requirements. In all other schools, parents were informed of the study and asked to contact the school or research team if they did not want their child to participate. Annual assessments were administered via a confidential online survey. Participant flow and retention rates for each condition are shown in Figure 1.

### Procedure

Schools were randomly assigned 1:1 to intervention or control conditions, matching on language and sex.^31^ At baseline, students completed the Substance Use Risk Profile Scale,^55^ a 23-item questionnaire that assesses 4 personality risk factors for substance use and mental health problems: anxiety sensitivity, hopelessness, impulsivity, and sensation seeking. Students scoring more than 1 SD above their school mean on any of the Substance Use Risk Profile personality subscales were allocated to the personality group on which they were furthest from the school mean.^14^ School-based means were used rather than population norms because personality norms for this age group and setting were not yet established and because baseline assessments were conducted on a rolling basis throughout the year, making it impractical to use the overall sample mean and SD for screening. This approach allowed us to identify youths at elevated risk relative to their peers within each school.

### Interventions

In schools randomized to the intervention group, students were invited to participate in the PreVenture program according to their allocated personality group. This intervention consists of two 90-minute group sessions, facilitated by a school-based counsellor during school hours. Sessions, delivered separately by personality group, included learning trait-focused case scenarios and cognitive behavioral strategies. Workshops are guided by facilitators who are trained to use motivational interviewing and core psychological counselling and cognitive-behavioral therapy principles tailored to the prevention context. Facilitators were trained to deliver the intervention in a 3-day workshop and received a minimum of 4 hours of supervision by the research term.^31^ Fidelity was assessed via an established measure of adherence to the core components of this intervention, and each facilitator was observed for at least 1 intervention session.^31^

The first session provided trait-specific psychoeducation on the cognitive-behavioral model of substance use (eg, catastrophic thinking and avoidance in the anxiety sensitivity group). In the second session, students learned to identify and challenge personality-specific thought patterns that contribute to problematic emotional and behavioral responses (eg, the sensation seeking group focused on addressing thoughts related to reward-seeking and boredom susceptibility).

Control schools delivered alcohol and other drug education, as usual. Further details about this intervention and the trial design can be found elsewhere.^24,31^

### Measures

Substance use was assessed using a modified and validated version of the Detection of Alcohol and Drug Problems in Adolescents.^56,57^ Participants reported their annual frequency of use of alcohol, cannabis, tobacco smoking, and nonmedical use of opioids (eg, codeine, meperidine hydrochloride, morphine, oxycodone, methadone, dextropropoxyphene, opium, hydromorphone, pentazocine) on a 6-point scale (0 indicates never; 1, occasionally; 2, approximately once a month; 3, weekends or once or twice during the week; 4, 3 times or more a week but not every day; 5, every day).

Illicit substances included cocaine, methamphetamine, ecstasy, heroin, lysergic acid diethylamide, phencyclidine, glue, and nonmedical use of prescription opioids, sedatives, or tranquilizers. Consistent with prior studies,^58^ participants reporting monthly use of 2 or more illicit substances were classified as illicit polysubstance users (0 indicating not a polysubstance user; 1, polysubstance user).

### Statistical Analysis

Analyses were conducted on an intention-to-treat basis using bayesian multilevel mixed-effect regression models to examine intervention effects over time. Models included random intercepts for individuals and schools to account for repeated measures and clustering. Time was coded as years from baseline (0, 1, 2, 3, and 4) and treated as continuous.

Frequency outcomes were modeled as ordered categorical variables and using bayesian cumulative ordinal regression with flexible thresholds—the recommended approach for outcomes with ordered but unequally spaced response categories.^59,60^ Polysubstance use was modeled via bayesian logistic regression (Bernoulli distribution). Linear growth and quadratic growth were compared using leave-1-out cross-validation, with lower expected log predictive density indicating better predictive accuracy.

An intervention-by-year interaction tested the intervention effects over time. Estimates were considered statistically credible if 95% credible intervals (CrIs) excluded zero.^59,61^ Effect sizes are reported as odds ratios (ORs), calculated by exponentiating the regression estimate. These odds ratios are individual-specific (ie, conditional on the random effects), meaning they describe the expected change in odds within a given individual or school, rather than averaged across the entire population. For ordinal outcomes, ORs reflect the annual change in the growth in the likelihood of participants being in a higher frequency category of substance use, compared with all lower categories.^62,63^ An OR less than 1 for the linear intervention-by-time interaction indicates a slower increase (or greater decrease) in the outcome over time in the intervention group relative to the control group. Sex differences in intervention effectiveness were explored 3 three-way interaction models (intervention × year × sex) for outcomes where credible group-by-year intervention effects were observed. Analyses were conducted using R version 4.4.1 (R Project for Statistical Computing), using the brms version 2.22.0 and loo version 2.8.0 packages from January to March 2025.

## Results

### Baseline Characteristics and Attrition

Of 3861 students at 31 schools screened, the intention-to-treat sample included 1669 students (mean [SD] age, 12.83 [0.47] years; 847 [50.7%] female) with elevated levels on at least 1 of the 4 target personality traits. Overall, 16 schools with 964 students were randomized to the control group and 15 schools with 705 students were randomized to the intervention group. Students in the control group were younger than students in the intervention group; and there were more students attending French-speaking schools in the control condition than the intervention condition (Table 1). Raw frequencies of outcomes over time are provided in eTable 1 in Supplement 2. Most participants completed at least 1 follow-up (1500 participants [89.81%]). Attrition analyses appear in eTable 2 and eTable 3 in Supplement 2. After adjusting for school-level variance, attrition neither differed between intervention and control conditions nor varied in relation to other baseline characteristics. As school-level variance is accounted for in all analyses, it was unnecessary to include additional covariates.

**Table 1.** Baseline Characteristics

Characteristic	Participants, No. (%)		
	Overall (N = 1669)	Control (n = 964)	Intervention (n = 705)
Age at baseline, mean (SD), y	12.83 (0.47)	12.81 (0.47)	12.85 (0.47)
Sex			
Male	822 (49.3)	468 (48.5)	354 (50.2)
Female	847 (50.7)	496 (51.5)	351 (49.8)
Language			
English	556 (33.3)	293 (30.4)	263 (37.3)
French	1113 (67.7)	671 (69.6)	442 (62.7)
Personality group			
Anxiety sensitivity	447 (26.8)	253 (26.2)	194 (27.5)
Impulsivity	395 (23.7)	230 (23.9)	165 (23.4)
Hopelessness	380 (22.8)	218 (22.6)	162 (23.0)
Sensation seeking	447 (26.8)	263 (27.3)	184 (26.1)

### Intervention Effects

Results from leave-1-out cross-validation model comparisons appear in eTable 4 in Supplement 2. Alcohol frequency and illicit polysubstance use exhibited linear growth while cannabis, tobacco smoking, and nonmedical opioid use frequency exhibited quadratic growth patterns. Including the interaction between intervention and the quadratic function of time (year-squared) did not improve predictive accuracy (and occasionally did not converge well); thus, this interaction term was excluded, and analyses focused on the interaction between linear effects of time and intervention.

**Figure 2.**
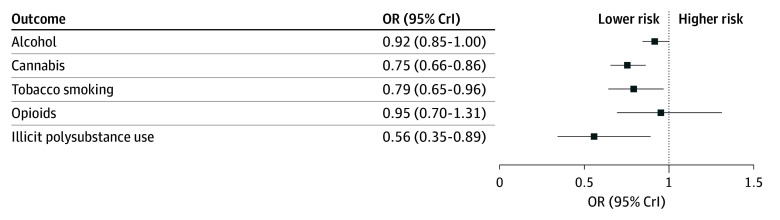
Intervention by Year Interaction Effects From the Final Multilevel Bayesian Regression Models CrI indicates credible interval; OR, odds ratio.

Results from Bayesian multilevel logistic and ordinal regression models are presented in Table 2, and ORs are displayed in Figure 2. Significant increases were observed in all outcomes over time.

**Table 2.** Bayesian Estimates of the Effect of the Intervention and Covariates on Substance Use Frequency Outcomes

Measure	*b* (SE) [95% CrI]
**Alcohol frequency**	
Intervention	0.36 (0.41) [−0.48 to 1.17]
Year	0.92 (0.03) [0.86 to 0.98]
Female sex	−0.18 (0.14) [−0.45 to 0.09]
French language	0.84 (0.38) [0.11 to 1.59]
Intervention × year	−0.09 (0.04) [−0.17 to −0.00]^a^
**Cannabis frequency**	
Intervention	0.98 (0.65) [−0.30 to 2.27]
Year	2.04 (0.13) [1.78 to 2.29]
Year-squared	−0.16 (0.03) [−0.21 to −0.11]
Female sex	−0.68 (0.22) [−1.11 to −0.26]
French language	0.52 (0.58) [−0.58 to 1.71]
Intervention × year	−0.28 (0.07) [−0.42 to −0.15]
**Tobacco smoking frequency**	
Intervention	0.55 (0.32) [−0.09 to 1.18]
Year	0.58 (0.19) [0.23 to 0.95]
Year-squared	−0.08 (0.04) [−0.15 to 0.00]
Female sex	−0.26 (0.17) [−0.60 to 0.06]
French language	0.05 (0.22) [−0.41 to 0.48]
Intervention × year	−0.23 (0.10) [−0.44 to −0.04]
**Illicit polysubstance use**	
Intercept	−7.48 (0.94) [−9.53 to −5.85]
Intervention	1.50 (0.73) [0.12 to 2.98]
Year	0.53 (0.17) [0.22 to 0.89]
Female sex	−0.74 (0.38) [−1.52 to −0.04]
French language	0.10 (0.51) [−0.82 to 1.17]
Intervention × year	−0.58 (0.24) [−1.06 to −0.12]
**Opioid frequency**	
Intervention	0.19 (0.65) [−1.10 to 1.47]
Year	1.48 (0.32) [0.89 to 2.12]
Year-squared	−0.19 (0.06) [−0.31 to −0.06]
Female sex	−1.02 (0.27) [−1.55 to −0.50]
French language	0.54 (0.47) [−0.37 to 1.51]
Intervention × year	−0.05 (0.16) [−0.36 to 0.27]

Abbreviations: CrI, credible interval; OR, odds ratio.

^a^ Unrounded upper CrI, −0.004.

#### Alcohol Use

Students in the intervention group had a slower rate of increase in the frequency of alcohol use over time (*b* = −0.09, 95% CrI, −0.17 to −0.00), reflecting a small negative effect (unrounded upper CrI, −0.004). Within-person annual growth in the odds of being in a higher frequency category of alcohol use was 8% slower in the intervention group than the control group (OR, 0.92; 95% CrI, 0.85-100).

#### Cannabis Use

Compared with the control group, students in the intervention group had a slower rate of growth in the frequency of cannabis use over time (*b* = −0.28; 95% CrI, −0.42 to −0.15). The annual rate of increase in odds of being in a higher frequency category of cannabis use was 25% slower in the intervention group than the control group (OR, 0.75; 95% CrI, 0.66-0.86).

#### Tobacco Smoking

Compared with the control group, students in the intervention group had a slower rate of growth in the frequency of smoking tobacco (*b* = −0.23, 95% CrI, −0.44 to −0.04). The annual increase in the odds of being in a higher category of frequency of smoking were 21% slower in the intervention group than the control group (OR, 0.79; 95% CrI 0.65-0.96).

### Illicit Polysubstance Use

Compared with the control group, students in the intervention group had a slower rate of increase in the odds of polysubstance use (*b* = −0.58; 95% CrI, −1.06 to −0.12), suggesting the annual increase in odds of illicit polysubstance use was 44% slower for participants in the intervention group (OR, 0.56; 95% CrI, 0.35-0.89). As the prevalence of polysubstance use was low, we conducted an additional analysis examining effects on the number of illicit substances used (eTable 5 in Supplement 2). This analysis found that students in the intervention group had a slower increase in the number of illicit substances used than the control group (*b* = −0.34; 95% CrI, −0.58 to −0.09).

#### Opioids

We observed a small reduction in the frequency of opioid use in the intervention group compared with the control group; however, there was no credible evidence for an intervention-by-time interaction (*b* = −0.05; 95% CrI, −0.36 to 0.27; OR, 0.95; 95% CI, 0.70 to 1.31). The direction of effect suggested that the intervention may have modestly attenuated increases in opioid use, but this effect remains uncertain.

### Sex Differences

In an exploratory post hoc analysis using bayesian regression models with 3-way interactions among intervention, year, and sex, there was no credible evidence for 3-way interactions for frequency of alcohol, cannabis, tobacco smoking, or illicit polysubstance use (Table 3). Thus, the effect of intervention on these outcomes did not differ by sex, suggesting the impact of the intervention on slowing increases in alcohol, cannabis, smoking, and polysubstance use was similar for both males and females.

**Table 3.** Bayesian Estimates for the Effect of the Intervention and Sex on Growth in Substance Use

Measure	*b* (SE) [95% CrI]	OR (95% CrI)
**Alcohol frequency**		
Intervention	0.17 (0.44) [−0.68 to 1.04]	1.19 (0.51 to 2.83)
Female sex	−0.44 (0.21) [−0.84 to −0.04]	0.64 (0.43 to 0.96)
Year	0.89 (0.04) [0.81 to 0.98]	2.44 (2.25 to 2.66)
French language	0.82 (0.36) [0.11 to 1.55]	2.27 (1.12 to 4.71)
Intervention × sex	0.36 (0.33) [−0.28 to 1.00]	1.43 (0.76 to 2.72)
Intervention × year	−0.12 (0.06) [−0.24 to 0.01]	0.89 (0.79 to 1.01)
Sex × year	0.05 (0.05) [−0.06 to 0.16]	1.05 (0.94 to 1.17)
Intervention × sex × year	0.05 (0.09) [−0.12 to 0.22]	1.05 (0.89 to 1.25)
**Cannabis frequency**		
Intervention	1.15 (0.71) [−0.26 to 2.53]	3.17 (0.77 to 12.55)
Female sex	0.09 (0.36) [−0.61 to 0.80]	1.10 (0.54 to 2.23)
Year	2.20 (0.14) [1.93 to 2.47]	9.04 (6.89 to 11.82)
Year-squared	−0.16 (0.03) [−0.21 to −0.11]	0.85 (0.81 to 0.90)
French language	0.51 (0.58) [−0.58 to 1.67]	1.66 (0.56 to 5.31)
Intervention × sex	−0.39 (0.54) [−1.42 to 0.69]	0.68 (0.24 to 1.99)
Intervention × year	−0.36 (0.10) [−0.56 to −0.17]	0.69 (0.57 to 0.84)
Sex × year	−0.31 (0.09) [−0.49 to −0.13]	0.73 (0.61 to 0.88)
Intervention × sex × year	0.16 (0.14) [−0.11 to 0.43]	1.18 (0.90 to 1.54)
**Tobacco smoking frequency**		
Intervention	0.80 (0.45) [−0.09 to 1.67]	2.21 (0.91 to 5.31)
Female sex	0.62 (0.40) [−0.17 to 1.42]	1.86 (0.84 to 4.14)
Year	0.75 (0.20) [0.37 to 1.13]	2.11 (1.45 to 3.10)
Year-squared	−0.07 (0.04) [−0.15 to 0.00]	0.93 (0.86 to 1.00)
French language	0.05 (0.23) [−0.41 to 0.48]	1.05 (0.66 to 1.62)
Intervention × sex	−0.60 (0.61) [−1.82 to 0.56]	0.55 (0.16 to 1.75)
Intervention × year	−0.35 (0.15) [−0.64 to −0.06]	0.71 (0.53 to 0.94)
Sex × year	−0.37 (0.14) [−0.64 to −0.11]	0.69 (0.53 to 0.90)
Intervention × sex × year	0.26 (0.21) [−0.14 to 0.67]	1.30 (0.87 to 1.95)
**Illicit polysubstance use**		
Intervention	2.24 (0.93) [0.48 to 4.15]	9.38 (1.62 to 63.43)
Female sex	0.09 (1.04) [−1.94 to 2.19]	1.10 (0.14 to 8.94)
Year	0.66 (0.24) [0.21 to 1.14]	1.93 (1.23 to 3.13)
French language	0.09 (0.53) [−0.87] to 1.23	1.09 (0.42 to 3.42)
Intervention × sex	−1.99 (1.45) [−4.92 to 0.76]	0.14 (0.01 to 2.14)
Intervention × year	−0.83 (0.31) [−1.45 to −0.24]	0.44 (0.23 to 0.79)
Sex × year	−0.24 (0.34) [−0.91 to 0.42]	0.79 (0.40 to 1.52)
Intervention × sex × year	0.63 (0.51) [−0.36 to 1.63]	1.87 (0.70 to 5.10)

Abbreviations: CrI, credible interval; OR, odds ratio.

## Discussion

This secondary analysis of a large-scale cluster RCT found that this personality-targeted brief cognitive-behavioral interventions effectively reduced the escalation of alcohol, cannabis, tobacco smoking, and illicit polysubstance use during adolescence, with similar effects for male and female students. These findings align with prior evidence showing that such interventions reduce substance use escalation during adolescence^28,29^ and extend this research by exploring effects on polysubstance use, nonmedical use of prescription opioids, and sex differences. Previous research in this cohort found the PreVenture program was associated with reduced odds of developing a substance use disorder.^24^ The current findings suggest that personality-targeted prevention can slow the rate at which adolescents increase the frequency of their substance use over time. Attenuating escalation in use might be a key mechanism for preventing substance use disorders among youths. Given the gradient of effect between frequency of use and negative outcomes, including drug fatalities,^3,6,33,64^ these findings underscore the value of personality-targeted approaches in preventing harmful substance use and disorder by reducing recurrent substance use and polysubstance use in adolescents.

The uncertain intervention effect on nonmedical use of prescription opioids may reflect the young age of participants, whose decision-making and susceptibility to social pressures are still developing, and the limited program content directly addressing prescription drug use. Nonmedical use of prescription drugs is less common during adolescence and typically onsets in emerging adulthood.^33,65,66^ However, the observed reduction in polysubstance use within this study included nonmedical use of prescription opioids, suggesting that the intervention may be effective in reducing opioid use as part of a broader polysubstance use pattern. Future adaptations of this intervention may consider enhancing effectiveness by addressing prescription drug use more explicitly and targeting older adolescents or young adults using appropriate measures of harmful use (eg, dependence), particularly given recent findings indicating a paradoxical trend of declining opioid use but rising opioid-related harms and dependence among youths.^10^ More broadly, future adaptations could consider incorporating contemporary social-contextual components to better equip adolescents to navigate the evolving social and digital environments that influence substance use behaviors.^65,66^

The lack of sex-related differences in intervention effectiveness is consistent with treatment research, where males and females show similar responses to treatment despite differing patterns of substance use and comorbidities.^42,67^ The personalized nature of this intervention may mitigate any potential sex-related differences, similar to individualized treatment approaches.

### Limitations

This study has some limitations. First, although retention was lower than expected (approximately 65% at the final time point), which is not uncommon in trials of this size and duration, rates did not differ significantly between groups after accounting for school-level variance. Furthermore, bayesian multilevel models make use of all available data (while also accounting for individual trajectories and school-level clustering) and provide more conservative uncertainty estimates than frequentist approaches.^68,69^ Second, some potential ethnic and social confounding or moderating factors were not measured, limiting our ability to assess effectiveness across diverse sociocultural contexts. Furthermore, this study assessed sex but not gender identity. At the time of study design, large-scale studies rarely distinguished between sex and gender; thus, effects related to gender diversity could not be examined. Third, this study focused on the main secondary outcomes and did not include trait-specific analyses. Future studies may examine the role of the intervention in previously identified personality-specific pathways to patterns of substance use via changes in mental health symptoms.^34^ Furthermore, as the study was conducted leading up to the legalization of recreational cannabis use in Canada, it is unclear whether similar effects would be observed following legalization.

## Conclusions

This secondary analysis of a cluster RCT demonstrated that a selective, personality-targeted prevention program reduced the escalation of substance use during adolescence, a critical developmental period when substance use patterns are established. Notably, the personality-targeted program was associated with a 25% lower rate of increase in cannabis use and 44% lower rate of increase in polysubstance use. Findings highlight the importance of early, targeted intervention approaches to mitigate the rising rates of substance use–related harms.^11,12,13^ Future research should investigate longer-term outcomes, explore potential moderators to optimize intervention, and consider evaluating this intervention’s impact on opioid use when delivered later in adolescence or in emerging adulthood.

## Notes

Supplement 1.
Trial Protocol and Statistical Analysis Plan

Supplement 2. **eTable 1.** Substance use outcomes at all measurement occasions **eTable 2.** Baseline differences between those who missed any follow ups and those present for all follow ups adjusting for school-level variance using multilevel logistic regression models **eTable 3.** Baseline differences between those who missed all follow ups and those present for at least one follow up adjusting for school-level variance using multilevel logistic regression models **eTable 4.** Model comparison of substance use trajectories: LOO-CV fit indices with linear and quadratic effects **eTable 5.** Bayesian estimates, standard errors, credible intervals and odds ratios for the effect of the intervention on the count of illicit substances used

Supplement 3.
Data Sharing Statement

## References

[1] Erskine HE, Moffitt TE, Copeland WE, . A heavy burden on young minds: the global burden of mental and substance use disorders in children and youth. Psychol Med. 2015;45(7):1551-1563. doi:10.1017/S0033291714002888 25534496 PMC5922255

[2] Whiteford HA, Degenhardt L, Rehm J, . Global burden of disease attributable to mental and substance use disorders: findings from the Global Burden of Disease Study 2010. Lancet. 2013;382(9904):1575-1586. doi:10.1016/S0140-6736(13)61611-6 23993280

[3] Danpanichkul P, Duangsonk K, Díaz LA, . The burden of alcohol and substance use disorders in adolescents and young adults. Drug Alcohol Depend. 2025;266:112495. doi:10.1016/j.drugalcdep.2024.112495 39603063

[4] Waller R, Murray L, Shaw DS, Forbes EE, Hyde LW. Accelerated alcohol use across adolescence predicts early adult symptoms of alcohol use disorder via reward-related neural function. Psychol Med. 2019;49(4):675-684. doi:10.1017/S003329171800137X 29871712 PMC7066874

[5] Lu W, Lopez-Castro T, Vu T. Population-based examination of substance use disorders and treatment use among US young adults in the National Survey on Drug Use and Health, 2011-2019. Drug Alcohol Depend Rep. 2023;8:100181. doi:10.1016/j.dadr.2023.100181 37593411 PMC10430156

[6] Degenhardt L, Stockings E, Patton G, Hall WD, Lynskey M. The increasing global health priority of substance use in young people. Lancet Psychiatry. 2016;3(3):251-264. doi:10.1016/S2215-0366(15)00508-8 26905480

[7] Jordan CJ, Andersen SL. Sensitive periods of substance abuse: early risk for the transition to dependence. Dev Cogn Neurosci. 2017;25:29-44. doi:10.1016/j.dcn.2016.10.004 27840157 PMC5410194

[8] Han B, Compton WM, Blanco C, DuPont RL. National trends in substance use and use disorders among youth. J Am Acad Child Adolesc Psychiatry. 2017;56(9):747-754.e3. doi:10.1016/j.jaac.2017.06.011 28838579

[9] Keyes KM, Kaur N, Kreski NT, . Temporal trends in alcohol, cannabis, and simultaneous use among 12th-grade U.S. adolescents from 2000 to 2020: differences by sex, parental education, and race and ethnicity. Alcohol Clin Exp Res. 2022;46(9):1677-1686. doi:10.1111/acer.14914 36125706 PMC9635013

[10] Jones CM. The paradox of decreasing nonmedical opioid analgesic use and increasing abuse or dependence - An assessment of demographic and substance use trends, United States, 2003-2014. Addict Behav. 2017;65:229-235. doi:10.1016/j.addbeh.2016.08.027 27561431

[11] Smith BT, Schoer N, Sherk A, Thielman J, McKnight A, Hobin E. Trends in alcohol-attributable hospitalisations and emergency department visits by age, sex, drinking group and health condition in Ontario, Canada. Drug Alcohol Rev. 2023;42(4):926-937. doi:10.1111/dar.13629 36843065

[12] Myran DT, Pugliese M, McDonald AJ, . Cannabis use disorder emergency department visits and hospitalizations and 5-year mortality. JAMA Netw Open. 2025;8(2):e2457852-e2457852. doi:10.1001/jamanetworkopen.2024.57852 39913138 PMC11803479

[13] Miech RA, Johnston LD, Patrick ME, O’Malley PM, Bachman JG. Monitoring the Future national survey results on drug use, 1975-2023: overview and detailed results for secondary school students. Institute for Social Research; 2023.

[14] Castellanos-Ryan N, Conrod P. Personality and substance misuse: evidence for a four-factor model of vulnerability. In: Verster JC, Brady K, Galanter M, Conrod P, . Drug Abuse and Addiction in Medical Illness. Springer Science + Business Media; 2012:47-62. doi:10.1007/978-1-4614-3375-0_4

[15] Conrod PJ. Personality-targeted interventions for substance use and misuse. Curr Addict Rep. 2016;3(4):426-436. doi:10.1007/s40429-016-0127-6 27909645 PMC5110575

[16] Conrod PJ, Castellanos N, Mackie C. Personality-targeted interventions delay the growth of adolescent drinking and binge drinking. J Child Psychol Psychiatry. 2008;49(2):181-190. doi:10.1111/j.1469-7610.2007.01826.x 18211277

[17] Conrod PJ, O’Leary-Barrett M, Newton N, . Effectiveness of a selective, personality-targeted prevention program for adolescent alcohol use and misuse: a cluster randomized controlled trial. JAMA Psychiatry. 2013;70(3):334-342. doi:10.1001/jamapsychiatry.2013.651 23344135

[18] Debenham J, Grummitt L, Newton NC, . Personality-targeted prevention for adolescent tobacco use: three-year outcomes for a randomised trial in Australia. Prev Med. 2021;153:106794. doi:10.1016/j.ypmed.2021.106794 34508734

[19] Edalati H, Conrod PJ. A review of personality-targeted interventions for prevention of substance misuse and related harm in community samples of adolescents. Front Psychiatry. 2019;9(JAN):770. doi:10.3389/fpsyt.2018.00770 30723431 PMC6349726

[20] Lammers J, Goossens F, Conrod P, Engels R, Wiers RW, Kleinjan M. Effectiveness of a selective alcohol prevention program targeting personality risk factors: Results of interaction analyses. Addict Behav. 2017;71:82-88. doi:10.1016/j.addbeh.2017.02.030 28282524

[21] Newton NC, Stapinski L, Teesson M, . Evaluating the differential effectiveness of social influence and personality-targeted alcohol prevention on mental health outcomes among high-risk youth: a novel cluster randomised controlled factorial design trial. Aust N Z J Psychiatry. 2020;54(3):259-271. doi:10.1177/0004867419877948 31561712

[22] Perrier-Ménard E, Castellanos-Ryan N, O’Leary-Barrett M, Girard A, Conrod PJ. The impact of youth internalising and externalising symptom severity on the effectiveness of brief personality-targeted interventions for substance misuse: a cluster randomised trial. Addict Behav. 2017;75(April):138-144. doi:10.1016/j.addbeh.2017.07.015 28734153

[23] Slade T, Newton NC, Mather M, . The long-term effectiveness of universal, selective and combined prevention for alcohol use during adolescence: 36-month outcomes from a cluster randomized controlled trial. Addiction. 2021;116(3):514-524. doi:10.1111/add.15178 32621555

[24] Conrod P, Stewart SH, Seguin J, . Five-year outcomes of a school-based personality-focused prevention program on adolescent substance use disorder: a cluster randomized trial. Am J Psychiatry. 2025;182(5):473-482. doi:10.1176/appi.ajp.20240042 39810554

[25] Boak A, Hamilton HA, Mann RE, Adlaf EM. Drug Use Among Ontario Students, 1977-2013: Detailed Findings from the Ontario Student Drug Use and Health Survey. Centre for Addiction and Mental Health; 2013. doi:10.1037/e506452014-001

[26] Boak A, Hamilton HA, Adlaf EM, Mann RE. Drug Use Among Ontario Students, 1977-2017: Detailed Findings from the Ontario Student Drug Use and Health Survey. Published 2017. Accessed April 13, 2025. https://www.camh.ca/-/media/files/pdf---osduhs/drug-use-among-ontario-students-1977-2017---detailed-findings-from-the-osduhs.pdf

[27] Champion KE, Debenham J, Teesson M, . Effect of a selective personality-targeted prevention program on 7-year illicit substance related outcomes: a secondary analysis of a cluster randomized controlled trial. Drug Alcohol Depend. 2024;258:111266. doi:10.1016/j.drugalcdep.2024.111266 38552600

[28] Conrod PJ, Castellanos-Ryan N, Strang J. Brief, personality-targeted coping skills interventions and survival as a non-drug user over a 2-year period during adolescence. Arch Gen Psychiatry. 2010;67(1):85-93. doi:10.1001/archgenpsychiatry.2009.173 20048226

[29] Mahu IT, Doucet C, O’Leary-Barrett M, Conrod PJ. Can cannabis use be prevented by targeting personality risk in schools: twenty-four-month outcome of the adventure trial on cannabis use: a cluster-randomized controlled trial. Addiction. 2015;110(10):1625-1633. doi:10.1111/add.12991 26011508 PMC5034824

[30] Newton NC, Teesson M, Mather M, . Universal cannabis outcomes from the Climate and Preventure (CAP) study: a cluster randomised controlled trial. Subst Abuse Treat Prev Policy. 2018;13(1):34. doi:10.1186/s13011-018-0171-4 30253790 PMC6157057

[31] O’Leary-Barrett M, Mâsse B, Pihl RO, Stewart SH, Séguin JR, Conrod PJ. A cluster-randomized controlled trial evaluating the effects of delaying onset of adolescent substance abuse on cognitive development and addiction following a selective, personality-targeted intervention programme: the Co-Venture trial. Addiction. 2017;112(10):1871-1881. doi:10.1111/add.13876 28544009

[32] Health Canada. Canadian Tobacco Alcohol and Drugs (CTADS): 2015 summary. Updated March 2017. Accessed April 9, 2025. https://www.canada.ca/en/health-canada/services/canadian-alcohol-drugs-survey/2015-summary.html

[33] Health Canada. Alcohol and drug use among students in Canada, 2023–24: key findings from the Canadian Student Alcohol and Drugs Survey. Updated March 2025. Accessed March 9, 2025. https://www.canada.ca/en/health-canada/services/canadian-student-tobacco-alcohol-drugs-survey/2023-2024-key-findings.html

[34] Stewart SH, Chinneck A, Thompson K, . Personality to prescription drug misuse in adolescents: testing affect regulation, psychological dysregulation, and deviance proneness pathways. Front Psychiatry. 2021;12:640766. doi:10.3389/fpsyt.2021.640766 33986700 PMC8110923

[35] Muhuri PK, Gfroerer JC, Davies MC. CBHSQ data review: associations of nonmedical pain reliever use and initiation of heroin use in the United States. Published August 2013. Accessed March 26, 2025. https://www.samhsa.gov/data/sites/default/files/DR006/DR006/nonmedical-pain-reliever-use-2013.htm

[36] Compton WM, Jones CM, Baldwin GT. Relationship between nonmedical prescription-opioid use and heroin use. N Engl J Med. 2016;374(2):154-163. doi:10.1056/NEJMra1508490 26760086 PMC11784537

[37] Konefal S, Sherk A, Maloney-Hall B, Young M, Kent P, Biggar E. Polysubstance use poisoning deaths in Canada: an analysis of trends from 2014 to 2017 using mortality data. BMC Public Health. 2022;22(1):269. doi:10.1186/s12889-022-12678-z 35144586 PMC8830122

[38] Compton WM, Valentino RJ, DuPont RL. Polysubstance use in the U.S. opioid crisis. Mol Psychiatry. 2021;26(1):41-50. doi:10.1038/s41380-020-00949-3 33188253 PMC7815508

[39] Elam KK, Mun CJ, Connell A, Ha T. Coping strategies as mediating mechanisms between adolescent polysubstance use classes and adult alcohol and substance use disorders. Addict Behav. 2023;139:107586. doi:10.1016/j.addbeh.2022.107586 36610287 PMC10075236

[40] Steinhoff A, Bechtiger L, Ribeaud D, Eisner MP, Quednow BB, Shanahan L. Polysubstance use in early adulthood: patterns and developmental precursors in an urban cohort. Front Behav Neurosci. 2022;15:797473. doi:10.3389/fnbeh.2021.797473 35153693 PMC8828938

[41] Nairn SA, Audet M, Stewart SH, . Interventions to reduce opioid use in youth at-risk and in treatment for substance use disorders: a scoping review. Can J Psychiatry. 2022;67(12):881-898. doi:10.1177/07067437221089810 35535396 PMC9659799

[42] McHugh RK, Votaw VR, Sugarman DE, Greenfield SF. Sex and gender differences in substance use disorders. Clin Psychol Rev. 2018;66:12-23. doi:10.1016/j.cpr.2017.10.012 29174306 PMC5945349

[43] Hawke LD, Koyama E, Henderson J. Cannabis use, other substance use, and co-occurring mental health concerns among youth presenting for substance use treatment services: sex and age differences. J Subst Abuse Treat. 2018;91:12-19. doi:10.1016/j.jsat.2018.05.001 29910010

[44] Schepis TS, Desai RA, Cavallo DA, . Gender differences in adolescent marijuana use and associated psychosocial characteristics. J Addict Med. 2011;5(1):65-73. doi:10.1097/ADM.0b013e3181d8dc62 21769049 PMC3359836

[45] Cooper ZD, Craft RM. Sex-dependent effects of cannabis and cannabinoids: a translational perspective. Neuropsychopharmacology. 2018;43(1):34-51. doi:10.1038/npp.2017.140 28811670 PMC5719093

[46] van Ours JC, Williams J. Why parents worry: initiation into cannabis use by youth and their educational attainment. J Health Econ. 2009;28(1):132-142. doi:10.1016/j.jhealeco.2008.09.001 18926578

[47] Charrier L, van Dorsselaer S, Canale N, . A Focus on Adolescent Substance Use in Europe, Central Asia and Canada: Health Behaviour in School-Aged Children International Report From the 2021/2022 Survey. Vol 3. World Health Organization; 2024.

[48] Cosma A, Elgar FJ, de Looze M, . Structural gender inequality and gender differences in adolescent substance use: a multilevel study from 45 countries. SSM Popul Health. 2022;19:101208. doi:10.1016/j.ssmph.2022.101208 36124256 PMC9482136

[49] Johnson RM, Fairman B, Gilreath T, . Past 15-year trends in adolescent marijuana use: differences by race/ethnicity and sex. Drug Alcohol Depend. 2015;155:8-15. doi:10.1016/j.drugalcdep.2015.08.025 26361714 PMC4582007

[50] Kuntsche E, Kuntsche S, Knibbe R, . Cultural and gender convergence in adolescent drunkenness: evidence from 23 European and North American countries. Arch Pediatr Adolesc Med. 2011;165(2):152-158. doi:10.1001/archpediatrics.2010.191 20921343 PMC4133118

[51] Vieno A, Lenzi M, Santinello M, Cavallo F. Gender convergence in adolescent drunkenness in different Italian regions. Int J Public Health. 2013;58(5):785-790. doi:10.1007/s00038-013-0447-4 23385392

[52] Lewis B, Hoffman LA, Nixon SJ. Sex differences in drug use among polysubstance users. Drug Alcohol Depend. 2014;145:127-133. doi:10.1016/j.drugalcdep.2014.10.003 25454410 PMC4254592

[53] Bonar EE, Coughlin L, Roche JS, . Prescription opioid misuse among adolescents and emerging adults in the United States: a scoping review. Prev Med. 2020;132:105972. doi:10.1016/j.ypmed.2019.105972 31904397 PMC7024638

[54] McHugh RK, Nguyen MD, Chartoff EH, Sugarman DE, Greenfield SF. Gender differences in the prevalence of heroin and opioid analgesic misuse in the United States, 2015-2019. Drug Alcohol Depend. 2021;227:108978. doi:10.1016/j.drugalcdep.2021.108978 34488078 PMC8516063

[55] Woicik PA, Stewart SH, Pihl RO, Conrod PJ. The Substance Use Risk Profile Scale: a scale measuring traits linked to reinforcement-specific substance use profiles. Addict Behav. 2009;34(12):1042-1055. doi:10.1016/j.addbeh.2009.07.001 19683400

[56] Bernard M, Bolognini M, Plancherel B, . French validity of two substance-use screening tests among adolescents: a comparison of the CRAFFT and DEP-ADO. J Subst Use. 2005;10(6):385-395. doi:10.1080/14659890412331333050

[57] Germain M, Guyon L, Landry M, Tremblay J, Brunelle N. Bergeron J. Grille de Dépistage de Consommation Problématique d’alcool et de Drogues Chez Les Adolescents et Les Adolescentes (DEP-ADO), Version 3.1. Accessed March 26, 2025. https://apimed-pl.org/contenu/uploads/2019/11/Grille-RISQ.2003-d%C3%A9pistage-alcool-drogues-ado.pdf

[58] Zuckermann AME, Williams G, Battista K, de Groh M, Jiang Y, Leatherdale ST. Trends of poly-substance use among Canadian youth. Addict Behav Rep. 2019;10:100189. doi:10.1016/j.abrep.2019.100189 31193263 PMC6525276

[59] Bürkner PC, Vuorre M. Ordinal regression models in psychology: a tutorial. Adv Methods Pract Psychol Sci. 2019;2(1):77-101. doi:10.1177/2515245918823199

[60] Bürkner PC. brms: an R package for bayesian multilevel models using Stan. J Stat Softw. 2017;80:1-28. doi:10.18637/jss.v080.i01

[61] Heino MTJ, Vuorre M, Hankonen N. Bayesian evaluation of behavior change interventions: a brief introduction and a practical example. Health Psychol Behav Med. 2018;6(1):49-78. doi:10.1080/21642850.2018.1428102 34040821 PMC8114380

[62] Cole SR, Allison PD, Ananth CV. Estimation of cumulative odds ratios. Ann Epidemiol. 2004;14(3):172-178. doi:10.1016/j.annepidem.2003.08.003 15036220

[63] McKinley TJ, Morters M, Wood JLN. Bayesian model choice in cumulative link ordinal regression models. Bayesian Anal. 2015;10(1):1-30. doi:10.1214/14-BA884

[64] Maggs JL, Calhoun BH, Allen HK. Substance use across adolescence and early adulthood: Prevalence, causes, developmental roots, and consequences. In: Crockett LJ, Carlo G, Schulenberg JE, . APA Handbook of Adolescent and Young Adult Development. American Psychological Association; 2023:541-556, . doi:10.1037/0000298-033

[65] Li X, Vaughn M, Xian H, Qian Z. Time spent on social media and the risk of substance use among US adolescents. J Adolesc. 2025;97(5):1314-1322. doi:10.1002/jad.12498 40188387

[66] Engel E, Gell S, Heiss R, Karsay K. Social media influencers and adolescents’ health: a scoping review of the research field. Soc Sci Med. 2024;340:116387. doi:10.1016/j.socscimed.2023.116387 38039770

[67] Volkow ND, Blanco C. Substance use disorders: a comprehensive update of classification, epidemiology, neurobiology, clinical aspects, treatment and prevention. World Psychiatry. 2023;22(2):203-229. doi:10.1002/wps.21073 37159360 PMC10168177

[68] Ma J, Thabane L, Kaczorowski J, . Comparison of Bayesian and classical methods in the analysis of cluster randomized controlled trials with a binary outcome: the Community Hypertension Assessment Trial (CHAT). BMC Med Res Methodol. 2009;9(1):37. doi:10.1186/1471-2288-9-37 19531226 PMC2703649

[69] Jones BG, Streeter AJ, Baker A, Moyeed R, Creanor S. Bayesian statistics in the design and analysis of cluster randomised controlled trials and their reporting quality: a methodological systematic review. Syst Rev. 2021;10(1):91. doi:10.1186/s13643-021-01637-1 33789717 PMC8015172

