# Supplementary material for: Selective Personality-Targeted Intervention and the Escalation of Substance Use During Adolescence: A Secondary Analysis of A Cluster-Randomized Clinical Trial
Source: JAMA Netw Open. 2025 Dec 18;8(12):e2550176. doi: 10.1001/jamanetworkopen.2025.50176 (PMC12715647; doi:10.1001/jamanetworkopen.2025.50176)
Supplement: Supplement 1. — Trial Protocol and Statistical Analysis Plan [file jamanetwopen-e2550176-s001.pdf]

CoVenture Trial Protocol and Statistical Analysis Plan

CoVenture Trial protocol: A cluster-radomised trial investigating the effects of selective intervention on adolescent cognitive development and addiction (September 07, 2018)

Statistical Analysis Plan for CoVenture Trial: A Cluster Randomized Trial Investigating The Effects Of Selective Intervention On Adolescent Cognitive Development And Addiction Version 1.1 (April 15, 2021)

Summary of Protocol Deviations, including deviation from planned statistical analyses (October 31, 2023)

1. The Need for a Trial

1.1 What is the problem to be addressed?

Adolescent onset alcohol and illicit drug use are associated with a myriad of immediate and long-term negative consequences [1]. Onset of alcohol use at or before 14 years of age is strongly associated with increased risk of developing alcohol use disorders, with rates of adult alcohol dependence in this early onset group estimated at 40% [2, 3]. Adolescent substance use is also associated with greater risk for mental health problems [4, 5], suicidal behaviour [6, 7], other drug use [3], poor academic performance [1, 8], school drop-out [9, 10], risky sexual behaviours [11, 12], poor physical health [13, 14], and injuries [15]. A recent World Health Organization study reported that alcohol use alone accounts for almost 4% of the global burden of health, with deaths attributed to alcohol greater than those caused by HIV/AIDS, violence or tuberculosis[16]. Moreover, a evaluation of drinking patterns in 73 countries worldwide reported that hazardous and harmful drinking patterns, such as drinking to intoxication and binge drinking, are on the rise among adolescents and young adults [17-19]. Compounding this problem are results from major epidemiological studies in the USA [20, 21] showing that the age of onset of alcohol use has been decreasing over the last 35 years, with youth now initiating alcohol use at 12 years of age on average. Statistics in Quebec are striking; 26.8% of 7th Grade students (12-13 year olds) report having used alcohol in the past year, almost half of whom (48.3%) have engaged in binge drinking during this period [22]. By grade 8, 46.8% of Quebec students have consumed alcohol, compared to 29.3% in the USA [20]. These results suggest that Quebec youth may initiate drinking earlier and show an accelerated growth in alcohol use, such that by the end of high school, 85.6% of Quebec students report alcohol use in the past year, 79.8% of whom binge drink[22]. The corresponding figures for the USA are 71% alcohol users by the end of high school, 30-60% of whom report binge drinking [20, 23, 24],despite being one year older than Quebec students. Illicit substance use is also very common amongst Quebec students (50%) and co-occurs with alcohol use [22]. It has been estimated that 10% of Quebec students suffer from a substance use disorder by the end of high school [22].

Brief, school-based coping skills interventions targeting personality risk factors for adolescent substance misuse have been shown to reliably delay onset of early substance use [25-28], effects which have been shown to last for up to two years in two separate randomised trials [26, 29]. As a primary goal, we now propose a longer-term trial of this intervention strategy to examine how this evidence-based intervention can reduce onset of substance use disorders in young people and related secondary mental health, academic and cognitive outcomes. As a secondary goal, we propose to use sensitive neuropsychological measures to examine how this evidence-based intervention can positively impact on cognitive development over the course of adolescence, to tease apart some of the mechanisms involved in the causal pathway from early onset substance use to poor cognitive development and long-term addiction outcomes.

Current theories on how early onset substance use impacts on future risk implicate the effects of alcohol and illicit substances on the adolescent developing brain. There is an extensive literature on the neuropsychological deficits in adolescents and adults with alcohol use disorders, and other substance use disorders. Cognitive impairments have been identified in multiple domains in adult alcoholics and drug users, including verbal and non-verbal performance, learning, memory, abstract reasoning, speed of information processing and efficiency [30-32]. These deficits have been replicated in adolescents with alcohol and substance use disorders, though on a smaller scale. Brown and colleagues [33] report a 10% weaker mental performance in alcohol-dependent 15-16 years olds relative to their nondrinking peers. Youth with alcohol use disorders were particularly impaired in tasks involving verbal or nonverbal memory recall, such as the Wechsler Memory Scales (WMS; [34]). Other studies have reported impairments in verbal and non-verbal memory, attention, executive and visuospatial performance [35, 36]. Sher et al [37] found differences in visuospatial ability (including WMS measures) and motor speed between groups of first-year college students with past year alcohol dependence relative to students with no past-year alcohol use disorder. This study showed that these alcohol-related deficits can be detected in young populations, even when controlling for other confounding factors such as family history of alcohol use disorders. Similarly,

spatial working memory deficits are found between alcohol-dependent women and control participants with no history of substance dependence aged 18-25 years [35].

Cognitive deficits have also been recognized in the non-problematic, social drinking population[38], with the suggestion that there is a continuum of deficits related to quantity of alcohol consumption. One of the most well-controlled investigations is a longitudinal study of neuropsychological functioning in adolescents assessed prior to initiating drinking and then over a 3-year follow-up, showing that those who transitioned into heavy or moderate drinking showed impaired cognitive function relative to their baseline levels and matched controls who remained nonusers throughout study [39]. Drinking days predicted a 10% reduction in visuospatial task performance from baseline to follow-up in girls and hangover symptoms predicted a 7% reduction in sustained attention for boys. Moderate to high levels of alcohol use and binge drinking may detrimentally affect neurocognitive development, and this study suggests that effects are detectable in the normal, social drinking youth population.

Adolescence represents a time of maturational change in the brain, and particularly the prefrontal cortex [40, 41]. The relatively late development of this area is thought to be associated with a salient increase in executive functioning and cognitive control capacity throughout adolescence [42, 43]. A number of studies have noted progressive linear changes between childhood and adulthood in task-specific (predominantly) prefrontal function during inhibitory and working memory functions [44, 45], and reward processing [46-48], with studies suggesting that adolescence is a unique point in development where inhibitory control is particularly dependent on incentive, particularly reward contingencies [46-48]. The former functions can be assessed using simple cognitive tasks such as Go/No-Go [49], which measure the ability to ignore and voluntarily suppress responses to task-irrelevant stimuli, or Self-Ordered Pointing tasks [50], which assess working and strategic memory as a proxy of executive function. Reward processing can be measured on similar tasks, but modified to include behaviour-dependent monetary reward or reward anticipation. Adults and adolescents with histories of substance use show abnormal behavioural and neural activation patterns on tasks of response inhibition and reward sensitivity [51-55]. These abnormalities have been shown to be exacerbated during substance withdrawal, and reduced reward-sensitivity has been shown to be restored following presentation of drug cues [56], suggesting that these abnormalities result, at least in part, from substance misuse and withdrawal, and might contribute to future addiction vulnerability. Adolescent brains may be particularly susceptible to damage from alcohol use due to the significant neuromaturation occurring throughout this period [1]. This has been shown to be true in rodent models, where alcohol-neurodegeneration is more severe in adolescent than adult brains [57]. Ethical considerations in human populations have precluded researchers from experimentally testing the same effect, but results have been mirrored to a certain extent in adolescent populations, where higher rates of nicotine or alcohol dependence are seen despite similar or lower levels of use than adults [40], suggesting heightened adolescent sensitivity.

Because the majority of neuropsychological studies with adolescents are cross-sectional, it has been difficult to conclude whether the observed cognitive abnormalities are causal or consequential to alcohol misuse. There is a large literature indicating that two major risk factors for adolescent onset alcohol misuse, namely family history of alcoholism and adolescent onset psychopathology [58], are associated with brain abnormalities that are also seen in adult substance abusers. Functions of inhibitory control, working memory, temporal foresight and delay of reward have been shown to be abnormal in children with disinhibited personalities, childhood disorders of impulsiveness, such as conduct disorder, attention deficit/hyperactivity disorder, and children of alcoholics [59-63]. Few studies are able to control for premorbid factors, but those that do show that alcohol-dependent youth with premorbid risk (e.g. familial alcoholism) show particularly impaired neuropsychological function [36], suggesting an interaction between vulnerability to substance misuse and the effects of substance misuse on the adolescent cognitive development. Current theories of adolescent brain development propose that it is adolescent developmental delay, rather than stable cognitive deficits, that account for the rise in risk taking and attentional difficulties in adolescence and the individual differences seen in these functions [40]. Therefore, longitudinal

designs that simply apply a pre-post design to control for baseline levels of neuropsychological function prior to onset of substance use might not capture individual differences in how the brain changes over the course of adolescence and how substance misuse and its growth might interfere with such development. Investigations involving multiple testing sessions and growth modelling analyses would be better suited to address this question.

Cannabis remains the most common illicit drug used throughout adolescence [20, 22], but there are fewer studies investigating its association with cognitive performance [64]. Adults with histories of heavy cannabis use show deficits in executive functioning [65, 66], and some studies show that early onset of cannabis use is associated with lower cognitive abilities later in life [67, 68]. As alcohol and other drug use often go hand in hand [3], we may therefore expect similar associations with neurocognitive functioning in adolescence.

The evidence reviewed suggest that early onset substance abuse is associated with neuropsychological impairment and future addiction risk. There is also some suggestion that adolescents might be more sensitive to the neuro-toxic effects of substances, which contributes to their addiction vulnerability. While questions remain pertaining to the extent to which cognitive precursors to early onset substance use account for these impairments, evidence from a few studies suggests that adolescent onset substance misuse produces some cognitive or emotional processing impairment beyond these premorbid deficits. The possible interaction between premorbid cognitive deficits and the effects of substances on cognitive development might also explain why early onset use so rapidly spirals into substance abuse and dependence as well as a myriad of other mental and phycial problems. Preventing early onset substance use could potentially have a broader effect on adolescent outcomes, including protecting adolescent cognitive development and future addiction.

1.2 What is/are the principal research question(s) to be addressed?

This proposal employs a unique opportunity to investigate the potential beneficial effects of alcohol and drug prevention on long-term onset of substance use disorders (primary) and the possible intermediary cognitive processes implicated in reducing such risk (secondary). A clinical trial with developmentally sensitive neuropsychological measures will examine whether preventing early onset substance use produces benefits on intermediate neuropsychological outcomes and longer-term, clinically-significant addiction outcomes.

We have shown that brief personality-targeted interventions that prevent or delay early onset of alcohol and illicit substance use in the short term (6 month) delay onset of more harmful substance use over the longer-term (up to 2 years post intervention), including onset of problem drinking symptoms [69, 70]. This suggests that there might be a critical period of development when preventing early use of substances could have longer-term effects on the development of substance use disorders. But we have yet to demonstrate effects of this intervention on clinically significant outcomes, which will be the **primary aim** of this trial. As a **secondary aim**, this new trial of a selective alcohol and drug prevention programme with established efficacy with respect to onset of substance use (previously shown to reduce rates of adolescent substance use by 30-80% up to 2 years post intervention [25-28]) will also incorporate a developmentally sensitive neuropsychological assessment to investigation of the effects of the intervention on cognitive outcomes which are presumed intermediate secondary outcomes in the causal pathway between early onset substance use and future addiction risk.

1. **Primary outcomes:**
    a. **Short-term:** delayed onset of alcohol and substance use (up to two years post intervention)
    b. **Long-term:** prevention of onset of substance use disorder (at 3 and 4-year follow-up).
2. **Secondary intermediate outcomes** are neuropsychological functions, for which two hypotheses will be investigated regarding the possible effects of delaying early substance use:
    a. Global Effects Hypothesis: substance use and binge drinking will have global harmful effects on cognition and interventions that successfully prevent substance use onset will result in global

improvements in cognitive function in participants who received the intervention relative to those randomized to control condition.

b. Critical Developmental Period Hypothesis: the toxic effects of alcohol and drug use are developmentally specific, so effects of interventions will be observed on cognitive processes that are maturing in adolescence, namely, executive functions and reward sensitivity, after controlling for general IQ and memory function following procedure described by Séguin, et al.[71].

3. **Secondary outcomes** will be measures of poor mental health and functional cognitive measures such as academic achievement and school drop-out.

1.3 Why is a trial needed now?

Historically, most school-based substance use prevention approaches have been universal, skills-enhancing programmes, with only mild and inconsistent effects on alcohol and drug use outcomes [72, 73]. Clearly new approaches to prevention are needed that translate knowledge from animal and human studies on addiction vulnerability. There is also a gap between the understanding of effective prevention strategies in the research context and school practice, with many schools using programs that are untested or ineffective [74]. The few evidence-based programmes that exist and show impact on variables beyond alcohol use [75] are only available in a small percentage of schools in North America or Europe. And two recent studies showed that the most evidence-based programme are associated with difficulties in implementation [76], and fidelity [77, 78].

One possible contributing factor to poor outcomes of universal prevention programmes is that they target generic factors implicated in normal drinking and drug use practices, and fail to target factors linked to individual differences in risk for the development of severe substance use problems [25-28]. Furthermore, difficulties in dissemination of effective programs might be related to the fact that outcome measures of substance use prevention trials have thus far not directly related to functioning in a school context and clinically significant outcomes. Schools' fundamental mandate remains to educate and maximise learning in their students [79], although this now includes a wide variety of health, social and culturally relevant areas [80, 81]. Thus, programs with outcomes that relate to more meaningful outcomes rather than just onset of substance use, such as learning, academic performance, drop-out and onset of disorder would be of particular interest to educational institutions and might encourage schools more to prioritise implementing evidence-based programmes [82].

Our research team has now repeatedly and consistently shown that psychological interventions targeting personality risk factors for substance misuse are highly effective in preventing and reducing alcohol and drug use in adolescents. Beneficial effects of the Preventure programme on substance use outcomes have been replicated in three separate trials in Canada [27] and the United Kingdom [25, 26, 28, 69], showing 30%-80% reductions in drinking, binge drinking and illicit drug use, and onset of problem symptoms over a two-year period. The Preventure programme has proven both feasible and effective when delivered by trained school-staff [28], and thus can operate within an implementation model that has a higher likelihood of being adopted by schools in a sustainable manner. We now aim to show impact of the programme on socially and clinically significant outcomes.

This study provides a unique opportunity to examine the beneficial effects of early intervention on cognitive development and future addiction, while controlling for neuropsychological factors implicated in the predisposition to early onset drinking. The outcomes of this trial are dually relevant for etiologic theories of addiction and for translating research to practice in real-world and sustainable ways.

1.4 Relevant systematic reviews

This trial is being proposed in light of evidence both on the effects of adolescent substance use on cognition and the dearth of prevention strategies effectively delaying early onset substance use/abuse. Spear [43] reviews evidence demonstrating the maturational changes and neural alterations taking place in the brain during adolescence, and the

increased pharmacological sensitivity to the effects of ethanol during this period of development. Crews and colleagues [83], refer to studies implicating adolescence as a period of critical cortical development in which disruption by alcohol and drug use has lifelong effects on cognition and behaviour. Clark et al [82] highlight the development during adolescence of prefrontal, limbic and reward circuits of the brain that affect individuals' cognitive behavioural and emotional regulation, and the dual vulnerability both to engagement in substance use behaviours as well as to the adverse effects of alcohol and drug exposure. Squeglia et al [84] review evidence on the effect of adolescent substance use on cognitive tasks in youth with as little as 1-2 years of heavy drinking, with impact on memory, attention, information processing, executive function and academic achievement. They suggest that heavy drinking during adolescence has subtle, but deleterious effects on cognitive functioning. Zeigler et al [1] highlight the immediate neurological consequences of acute intoxication during adolescence, where binge- drinking followed by abstinence mirrors the cognitive, physical and psychological symptoms documented in alcohol abusers undergoing withdrawal as well as the functional deficits seen in adolescent and adult alcohol users on a number of cognitive functions. Brown and Tapert [85] review the longevity of cognitive impairments resulting from adolescent alcohol use, with deterioration in functioning lasting into adulthood, as well as after abstinence from alcohol in individuals recovering from alcohol use disorders.

All of the reviews cited above conclude the need for more attention toward alcohol and drug prevention, raising the possibility that delaying substance use to a period of reduced vulnerability to toxic effects could protect cognitive development in adolescence and thus protect against future risk for addiction. Systematic reviews of school-based alcohol [73] and drug [72] prevention programmes conclude that the majority of such interventions are universal and have only mild or inconsistent effects. Other studies [86-89] have reviewed the characteristics of effective school-based programmes and conclude that targeting high-risk youth, interactive group sessions and peer involvement are components essential to maximising efficacy, which are components that have been incorporated in the Preventure programme. In light of studies showing reliable and consistent replicable efficacy [25-27] and effectiveness [28] of selective school-based prevention programme targeting those with personality risk factors for substance misuse, we are thus able to justify a longer-term trial to examine the resulting effects on cognitive development and addiction outcomes.

1.5 How will the results of this trial be used?

The results of this trial will be used to clarify the causal pathway between early onset substance use and cognitive development during adolescence, and whether delaying substance use onset can result in improvements in cognitive function and future reduction in risk for addiction. The experimental design of this trial will allow us to examine the course of cognitive development in high risk youth who are assisted in delaying their onset of alcohol use, providing for the first time, an opportunity to tease apart the causes of early onset substance use from the consequences of adolescent substance use from a cognitive-developmental perspective. This study will also allow us to examine how these possible cognitive consequences are implicated in future risk for addiction [82, 83], mental health problems [4, 5], poor academic performance [1, 8], and school drop-out [9, 10]. Findings will dramatically impact on public policy relating to youth substance use and the need for further attention toward prevention services in Canada. Cognitive outcomes relate directly to school-related measures, thus may increase interest in and likelihood of implementation of substance use prevention programmes in schools. Results may also increase interest within the school system, by parents and youth, should it be shown that cognitive function could be improved by targeting substance use risk.

1.6 Describe any risks to the safety of participants involved in the trial.

There is a risk to implementing interventions with youth, in that certain prevention programs have shown negative effects [90, 91], including consistent negative effects in non-cognitive-behavioural counselling and social work interventions [78]. However, the proposed personality-targeted interventions incorporate therapeutic techniques associated with beneficial outcomes [88] and have been shown to have substantial positive effects in three separate

populations across Canada [27] and the United Kingdom for individuals participating in interventions [25, 26, 28], as well as indirect population-level benefits across the school grade [29].

There are possible risks in implementation of selective interventions by educational professionals in a school context, both for adolescent participants and facilitators. The targeted nature of the programme necessitates a careful introduction of the study and interventions to participants in order to avoid participants being labelled or stigmatised. All self-report data will be kept confidential and parents and teachers consent to not having access to their child's self report information. Selection criteria are kept confidential and are only revealed to the staff members who are trained to deliver the programme and who undergo careful training on the importance of confidentiality around this programme. This programme works with educational professionals and school counsellors who are professionally trained to deal with issues of confidentiality, student distress and at risk cases. A clinical psychologist will be available to provide research staff and schools with additional clinical support should issues of risk be revealed. These concerns have been addressed and evaluated throughout the delivery the Adventure Trial, a teacher-delivered personality-targeted intervention trial [28], as well as during intervention delivery by psychologists [25-27, 69]. The proposed protocol is designed to minimise any risk to those involved.

2. The Proposed Trial

2.1 What is the proposed trial design? E.g. Open-label, double or single blinded, etc.

The study will use a cluster-randomised controlled design to test the effects of personality-targeted interventions on substance use and cognitive outcomes. Thirty-two schools (cluster) will agree to conduct annual surveys for 5 years with all consenting adolescents who are enrolled in Grade 7 in September 2012. Schools will be randomly assigned to 1) be trained to deliver the intervention programme and assisted in delivering the selective programme to this Grade 7 cohort or 2) assigned to receive training at the end of the trial, so that their corresponding Grade 7 cohort is never exposed to the intervention. Intervention schools will be assisted by research staff in organisation, delivery and evaluation of interventions delivered to their high risk students meeting criteria for personality risk. All schools will be assisted by our research team in arranging and implementing baseline and follow-up assessments at 12, 24, 36 and 48 months post-randomisation (Appendix B-Consort Figure).

This selective intervention involves screening all students in the first term of their Grade 7 year on personality dimensions linked to risk for substance misuse. Those scoring one standard deviation above the school mean on one of the following four dimensions will be invited to participate in coping skills workshops: Anxiety sensitivity, Hopelessness, Impulsivity, Sensation seeking. Due to the confidential nature of the selection criteria, this information will only be revealed to the teachers who deliver the training workshops. Feedback will not be provided to high risk youth or their parents.

This trial does not include a placebo condition, nor do we attempt to control for therapist contact in the control condition to avoid possible iatrogenic effects of an untested placebo intervention, which have been observed with youth interventions [90]. Furthermore, several systematic reviews of school-based interventions do not show natural reductions in youth substance use resulting from inactive group sessions.

This trial will be open-label, as it will be difficult to blind some participants (facilitators and selected students) to the fact that training and interventions are occurring in their schools. However, due to the confidential nature of the selection criteria for the programme, intervention assignment will be masked for unselected peers and teachers who are not directly involved in the delivery of the programme.

This five-year trial will allow us to observe intervention effects on the development of problematic substance use, which naturally progresses from 13 to 17 years of age and typically has an age of onset of first symptoms at 12-13 years. Similarly, linear changes in cognitive development can be detected over a 5-year period [46-48]. Annual

assessment of onset and severity of substance use and cognitive functioning will provide a sensitive design to examine causal hypotheses within the path from early prevention of substance use to cognitive outcomes and future risk for addiction. See Appendix C for study timeline.

2.2 What are the planned trial interventions? Both experimental and control.

The Preventure Programme is the first and only school-based alcohol and drug prevention programme that has been shown to prevent onset and growth in alcohol and substance misuse in British and Canadian youth [25-29]. Unlike universal programmes that tend to universally promote generic coping skills and balance normative attitudes around substance use, this selected personality-targeted approach is based on a etiologic model of addiction outlined and validated by the PI [27, 29, 92, 93], and targets four personality-specific motivational pathways to substance misuse: Hopelessness, Anxiety Sensitivity, Impulsivity and Sensation Seeking, each associated with different motives for substance use [93], drug use profiles [93, 94] and patterns of non-addictive psychopathology [95].

After selection on personality scales (see 2.5), high risk youth are assigned to personality-specific groups and are invited to participate in two 90-minute group sessions facilitated by a trained facilitator and co-facilitator. The interventions are conducted using manuals which incorporate psycho-educational, motivational enhancement therapy (MET, [96]) and cognitive-behavioural (CBT) components, and include real life 'scenarios' shared by local youth in with similar personality profiles. In the first session, participants are guided in a goal-setting exercise, designed to enhance motivation to change behaviour. Psycho-educational strategies are then used to teach participants about the target personality variable and associated problematic coping behaviours like avoidance, interpersonal dependence, aggression, risky behaviours and substance misuse. They are then introduced to the CBT model and guided in breaking down personal experience according to the physical, cognitive and behavioural components of an emotional response. A novel component to this intervention approach is the fact that all exercises discuss thoughts, emotions and behaviours in a personality-specific way, e.g. identifying situational triggers and cognitive distortions related to SS specifically. In the second session, participants are encouraged to identify and challenge personality-specific cognitive distortions that lead to problematic behaviours. For more information on the content and components of the intervention, refer to Appendix G and [25-29, 69].

Control participants will receive minimal drug education and this will depend on the school policy and curriculum. Information on other drug and alcohol prevention programmes delivered at the school and the extent to which participants in the study cohort received the intervention will be recorded. To incentivise control schools to remain in the trial, they will be provided with training on the delivery of the programme at later points in the trial, after the study cohort has completed their final outcome assessment in Grade 11.

Training will be offered to up to 4 staff-members per school. Trainees will include teachers, heads of year, school counsellors, and special educational needs staff who either volunteer or are nominated by the Principal to serve as a Preventure facilitator and school contact for the duration of the trial. They will then attend a 3-day workshop reviewing principles of CBT, MET and general counselling involving didactic instruction and role-play exercises. They will also be instructed on the theoretical basis of the programme, how to use the assessment/selection tool, and details of the research protocol, including the rationale and need for strict compliance around confidentiality, randomisation, voluntary participation and parental consent. Following the 3-day workshop, the trainees will receive a minimum of 4-hours supervision and feedback in running through a full intervention from the research therapist. Supervised interventions will be run with groups of Grade 7-8 students who are not involved in the formal trial. For more detail on training, supervision and accreditation of school facilitators see O'Leary-Barrett et al [28], and Appendix G.

2.3 What are the proposed practical arrangements for allocating participants to trial groups?

Randomisation of schools to intervention or control conditions will be on a 1:1 ratio using a restricted randomisation scheme, to match schools on various demographic characteristics linked to substance use. A staggered approach to randomisation, occurring following recruitment of every 10 schools will allow the clinical staff to stagger training and supervision over the course of the first year of the study. Dr. Masse and the staff at the Unité de recherché Clinique appliquée (URCA), Ste Justine, will oversee randomisation and will make sure that schools randomised to intervention and control will be matched on SES (private vs. public), size (number of grade seven classes), gender-specificity (mixed, girls-only, boys-only), and language (French vs. English). All students in grades 7 will be invited to take part in the trial, both survey and intervention phases and regardless of risk status. Responses on Substance Use Risk Profile Scale [93] will determine personality risk status (comparisons are made relative to school mean for each personality dimension). All HR students in intervention schools who consent to intervention phase of the study will be invited to take part in interventions delivered by trained school staff. All students, regardless of risk status, will be followed annually until Grade 11.

2.4 What are the proposed methods for protecting against sources of bias? E.g. Blinding or masking..

The trial coordinator and clinical psychologist overseeing training will not be blind to intervention allocation, but all trial staff who are involved in data acquisition will remain blind. Data will be anonymised; quality control of data will be conducted by staff blind to treatment condition.

This is not a fully double-blind study, but there will be some masking of school allocation and selection of participants. Schools are randomly assigned to intervention or control conditions, and a small number of school staff will be trained in the delivery of these interventions. Due to the targeted nature of the study, in order to avoid any potential effects of labelling attached to students participating in the intervention sessions, most school staff will be blind to the randomisation and risk status of students. Only trained facilitators in intervention schools will be provided with this information, which will be treated as confidential. In addition, all self-report data will be kept confidential and these facilitators will not be responsible for data collection. The general school staff will be informed that the whole year group are taking part in a research project and that some students will be invited to take part in coping skills interventions, however it will be emphasised that the interventions are meant to help youth channel their personality style towards their longer-term goals and will therefore not target students based on problem behaviours.

In order to maintain interest of control schools in this long trial, we will begin to train control schools in the delivery of the programme to subsequent Year 7 cohorts in the third year of this trial (after the study cohort have completed their 36 month assessment). This is the procedure that we used in the Adventure Trial, which did not appear to have any impact on the long-term results of the trial [29]. Training occurs over the course of the year in which 36-month outcomes are assessed at the schools, and schools will be provided with manuals and supervised in the delivery of the programme to the Year 7 cohort of 2016-17 after the final assessment of the study cohort in Year 11, creating a broader perception that the programme is being delivered in all schools.

Another important source of bias is lost to follow-up (LFUP), which can be related to primary and secondary outcomes. Most students drop out of a trial due to graduating from high school and moving to other schools, but this will not be a problem in this trial, as we do not expect to follow-up students beyond Grade 11. We also plan to use specific analytic methods, such as survival analysis, to manage this bias, which allow for assessment of outcome without necessarily requiring follow-up data at every point [26].

2.5 What are the planned inclusion/exclusion criteria?

Selection of schools: Inclusion criteria for schools will be that they have a Grade 7 cohort of at least 100 youth, providing access to 40-50 high risk students per school. Schools cannot be classified as having a majority of their

students coded as special needs students, because these schools are smaller and the intervention protocol would have to be tailored for their particular needs.

Selection of high risk youth: Inclusion criteria for students are that they be enrolled in Grade 7 and that they have provided active assent and passive parental consent to participate in the longitudinal survey and randomised trial phases of this study. Participants who score 1 standard deviation above the school mean on one of four subscales of the Substance Use Risk Profile Scale [93] will be selected to participate in the intervention groups, but all students will be followed on outcomes. There are no other exclusion criteria for participants.

2.6 What is the proposed duration of treatment period?

Outcomes from the Preventure and Adventure trials revealed that personality-targeted interventions delayed onset of drinking, binge drinking, problem drinking symptoms and illicit drug use in high-risk youth over 24-months [26]. The current trial will extend our outcome by two more years to determine if this early delay of onset of substance misuse translates to longer term outcomes on onset of substance use disorders, as according to the table presented in Appendix F on rates of substance misuse in Quebec youth, rates of problem alcohol or drug use will be detectable in youth at the grade 10 or grade 11 level. This long-term evaluation will allow interventions to occur in the year prior to the natural onset of substance use in the majority of Quebec youth, Grade 7-8.

2.7 What is the proposed frequency and duration of follow up? One baseline assessment and four annual follow-ups.

2.8 What are the proposed primary and secondary outcome measures?

The **primary outcomes** of this study are self-report alcohol and illicit drug use measures in high-risk youth, including onset of drinking, binge drinking and illicit drug use, within the first two years of the follow-up, and longer-term reductions in substance use disorders in later years, assessed according to a score greater than 20 on the 'Detection of alcohol and drug problems in adolescents' questionnaire (DEP-ADO [97]). This tool has demonstrated good construct validity, internal consistency, test-retest and intermodal execution reliability in Quebec youth. Self-report measures have been found to have excellent discriminant [98] and predictive [99] validity with regards to adolescent substance-related behaviours and problems [25-27, 69] This questionnaire captures quantity, frequency of drinking, binge drinking and illicit drug use, as well as symptoms of substance abuse and dependence, reliably identifying youth with alcohol use disorders [100].Youth who screen positive on this task will be contacted outside of the school setting to participate in a confidential phone or face-to-face interview, in which DEP-ADO will be administered by a clinician to confirm substance use disorder diagnosis.

Intermediate secondary outcomes are cognitive functioning. Global cognitive functioning, executive function and reward sensitivity will be assessed using neuropsychological measures relating to each of the hypotheses outlined in section 1.2. All cognitive tasks will be administered annually, during a 50-minute class period using computerised neuropsychological batteries developed, validated and shown to predict future academic and work performance by PI Peterson et al. [100] and physical aggression by PI Séguin et al. [71].

1. Global Effects Hypothesis will be tested using a computerised progressive matrices task [101] and a general knowledge task based on that used in [71], as proxies for global cognitive functioning. A pattern recognition task, a spatial recall task, verbal recall task and a visual reproduction recall task will be used to measure immediate and delayed visual and verbal memory, following Peterson et al. [100]. Similar cognitive scales have identified youth with alcohol use disorders [37].

2. Critical Developmental Period Hypothesis will be tested using two developmentally sensitive neuropsychological measures administered using the computerised battery of dorsolateral prefrontal tasks developed by Peterson et al [100]. One task, adapted from the Go/No-Go Passive Avoidance Learning Paradigm (PALP; [102]) will be used to assess cognitive control and response inhibition (see Appendix H). Commission errors on this task are commonly used as an index of response inhibition [103], and commission errors and reaction time to reward trials relative to punishment trials can be used as an index of reward sensitivity [52]. This task is

sensitive to individual differences in response inhibition under different reinforcement contingencies and with French and English-speaking adolescents and parallel forms exist for repeated administration [100, 103, 104]. The self-ordered pointing task [50] is a test of non-spatial executive working memory (Appendix H). This task is a sensitive measure of executive working memory in children [105], has been computerised and used to investigate working memory capacity in French and English-speaking adolescents at risk for substance misuse [71, 106].

Other secondary outcomes: Academic performance will be recorded by requesting access to students' school grades and provincial exam scores. School attendance, retention and drop-out will be requested from schools, with parent and student consent, data that 95% of schools were willing to provide in the Adventure RCT [28, 29]. Self-report mental health symptoms will be assessed by the Strengths and Difficulties Questionnaire [107], used in French-computerised format by the PI in the IMAGEN multi-site study of 2000 European 14-year olds [104]. Population level effects on substance use and cognitive outcomes will be examined to be sure that any benefits of the intervention on HR youth are not offset by possible negative or null effects on the non-targeted low-risk youth.

2.9 How will the outcome measures be measured at follow up?

Follow-up outcomes for self-report and cognitive measures will be assessed using the same measures as the initial assessment, in class sessions at 12-months intervals for 4 years. A computerised web-based data-acquisition platform will be used to collect self-report and cognitive data. Researchers will bring electronic Netbooks to classrooms and will assess whole grades of students at a time. Netbooks will allow us to avoid any scheduling difficulties associated with trying to use the school computer laboratories. Should students be absent from follow-up sessions, efforts will be made to organise additional data collection sessions at the school. Academic and attendance data will be requested from the school each year. Parallel forms of neuropsychological tasks will be used across repeated measurements, counterbalanced for order of presentation, to address potential learning effects, but most concerns around repeated assessment are addressed by random allocation to intervention groups. The data monitoring committee will pilot parallel tasks prior to data collection to detect possible ceiling effects.

In year 3 and in year 4 of follow up in data assessment, we will be asking the Ministry of Education their collaboration threw the ''Comité d'Accès à l'Information''to access the grades of all the participants in this study obtained on their ministry exams done in grade 9 and in grade 10. By having access to the ministry grades, we will be able to measure whether our intervention has a positive effect on child cognitive development since the ministry exams are associated to cognitive development in child development as well. Therefore we can demonstrate following our analyzes whether the intervention has a positive effect on the ministry grades by comparing them to the control group and thus promote the implementation of the program Preventure in schools and overlook this positive outcome. We have decided to use the ministry exams as a measure of outcome on cognitive development since it's an homogenous tool across our whole sample data.

Relatively to the other clinical data collected, all ministry grades of each participant will be kept locked in our files a for confidentiality and as well, they will be assigned to the participant by a research code that we will give to the school and the school will transmit to ministry of education, the date of birth, the permanent code and the research code of each participant.

2.10 Will health service research issues be addressed?

Formal health economic analyses will not be conducted, but cost of unit change in substance use will be calculated by calculating the cost to prevent onset of one case of substance use disorder at the final follow-up. A cost-effectiveness study as part of this RCT will be the subject of a separate application.

2.11 What is the proposed sample size and what is the justification?

Primary Analyses: Drinking outcomes at 12 and 24 months are considered established intermediate factors in the

causal pathway to substance use disorders. Primary outcomes of the current trial will focus on onset of substance use disorder at the 3 and 4-year follow-up (Grade 10 and 11), in the HR sample. Intercluster correlations were high for drinking (0.23-.27), but low for problem drinking (0.09) according to the Adventure Trial (Apprendix E). Power analyses were conducted according to Donner and Klar [108, 109]. According to the tables in Appendix F, the expected proportion of students demonstrating substance use disorder in grade 10 and 11 in the control group is 10% (but should be higher in the high risk sample). According to the two-year Adventure outcomes, we expect a 50% reduction from 20% to 10%, or conservatively from 10% to 5%. As well, we are expecting this proportion to vary between schools from 3% to 17%. Further assuming that the school proportions are approximately normally distributed where 95% of the schools would fall within two standard deviations of the mean (10% +- 7%), this leads to a coefficient of variation, k, of 0.35. With these assumptions and using formula 7.7 of White [110] one can obtain the number of schools (clusters) per group needed to detect a 50% reduction in the proportion (i.e. from 10% to 5%) with 80% power and a false positive error rate of 2.5%; 15 schools per group are needed for a total of 30 schools. Two additional schools will be recruited to allow for drop-out.

Secondary Objective and Cognitive Outcomes: According to analyses on the IMAGEN sample of 2000 European 14-year olds assessed on substance use and neuropsychological measures, binge drinkers showed reduced IQ and

greater reward bias on the PALP relative to non-bingers with effect sizes (d) for mean differences ranging from .23-.25, p=0.05 [106]. Larger differences are expected as the sample ages and substance use becomes more frequent. A conservative power analysis was conducted to be sure that we could detect effects on cognitive functioning at earlier ages. A sample size of 15 clusters per group with 50 HR adolescents per cluster achieves at least 90% power to detect a difference of at least 3.0 between the group means when the standard deviation is 15.0 (an effect size of 0.20) and the intra-cluster correlation is 0.40 using a false positive error rate of 2.5%.

2.12 What is the planned recruitment rate? How will the recruitment be organized? Over what time period?

We aim to recruit 32 schools between September 2011 and June 2012 (project to start September 2011). On the Island of Montreal, there are five major school boards servicing a total 72 secondary schools, and 80 large private secondary schools. Letters of support are provided from 3 of the 5 school boards and one of the largest private schools in the city. Our experience with the London-based Adventure Trial was that this number of schools could be recruited within the proposed recruitment time and expressions of interest to participate by schools far surpassed our recruitment needs. Letters of support from school boards and schools represent expressions of interest, but commitment to a formal research trial will be provided following ethical review of the project by the host institution and local school boards. Individual schools will be approached for participation after ethical approval is granted. One important incentive for schools is that all schools will be trained and supervised in the delivery of the program and provided with program materials at no cost. Should we experience difficulty recruiting 32 schools on the Island of Montreal, we will broaden our recruitment to the North and South Shores of Montreal, which will expand the list of potential high schools to over 250 schools.

In year 4 and year 5 of data collection, we also would like to solicit the participants in all our sample study for follow up after year 5 of assessment when they will be finishing high school and going off to CEGEP. We will be asking them to fill out a form with their coordinates and their approval for a member of our research team to contact them in the future to participant in other of our projects jointly held in our research laboratory.

2.13 Are there likely to be any problems with compliance? On what are the compliance figures based?

Compliance will be defined as being able to conduct all five assessments and, for intervention schools, being able to provide interventions to 85% of all high risk youth in their school. Based on previous studies, schools generally had very little difficulty complying and implementing the protocol, with only one school dropping out of the Adventure Trial due to systematic problems within the school and an impending school closure. Another intervention school was unable to complete the protocol for running sufficient interventions sessions with HR students and was thus not included in the trial. But the main objective of the Adventure Trial was to evaluate effectiveness of the Program, so research staff in this previous study could not offer additional support to schools if they could not deliver a certain aspect of the intervention protocol. Effectiveness will not be evaluated in the proposed study, and the current protocol will allow for research staff to assist schools in meeting demands of the research or intervention protocol, thus further improving compliance rates in the proposed trial. Based on recruitment rates from the three previous trials, student participation rates are anticipated as being >85%, and with a passive parental consent procedure, we should see very little attrition due to non-consent. One of the main sources of attrition in school-based research is requiring that parents provide active, signed consent for their child to participate. Passive consent was used at various stages in the three previous trials conducted by the PI and are easily justified to an ethics committee by our data suggesting that high risk youth are more likely to volunteer to participate in these preventative interventions, but less likely to be able to provide signed consent forms from their parents, likely due to personality and related cognitive difficulties in the child or the family.

2.14 What is the likely rate of loss to follow up? On what evidence is the loss to follow-up rate based?

This trial protocol is much based on that of the Adventure Trial, which achieved an average follow-up rate across two treatment conditions and four follow-up periods of 84% (including the 2 schools unable to complete follow-

up), with follow-up at the end of a two-year trial at 80%. We expect better follow-up in this trial, due to the less transient nature of a Montreal sample relative to a London sample, because participants will be recruited earlier in their academic career and required by law to be enrolled in school until the age of 16 years. Participation by school boards, rather than individual schools in London, will allow us to track students who transfer across schools while remaining in the trial. See Appendix D for recruitment and follow-up rates of the Adventure Trial[29].

2.15 How many centers will be involved? All recruitment will be conducted by CHU Ste Justine, U of Montreal, but University of Toronto and McGill will assist with data collection, management and analysis.

2.16 What is the proposed type of analyses?

Primary outcomes are on the intent to treat sample of youth who meet high risk (HR) personality criteria. To account for within cluster (school) variance, intervention effects will be examined using hierarchical generalized linear modelling (HGLM; using the program HLM 6) [111, 112] using a Bernoulli distribution for the binary primary outcome. Secondary outcome variables will be centred at the 12-month post-test to allow for comparisons between groups (intervention vs. control) after the intervention, and growth terms will be used to detect prevention effects on growth of onset of target behaviour over four years. For the three drinking outcomes, unconditional models from previous trials revealed that more than 10% of systematic variance existed at the between-school level (25-27%), therefore, HLM with robust standard errors will be used to assess intervention effects on the 12-month intercept and growth in drinking from 12-48 months [111]. When significant effects are revealed on growth, STATA (StataCorp, College Station, TX) will then be used to conduct logistic regression analyses testing intervention effects on onset of use at specific time points while controlling for cluster, using tests based on the Huber-White sandwich estimate of variance [110]. The illicit drug use outcome, problem drinking outcomes and cognitive outcomes are not expected to have high inter-class correlations [29]; HGLM will be used to conservatively assess these outcomes, nevertheless. We will also report cluster-level analyses in which individual-level variability is removed and analyses are conducted at the school-level, comparing mean outcomes for intervention (n=15) and control schools (N=15) using one-way repeated measures design with time as repeated measures and intervention as a fixed independent variable following recommendations by [113]. Path analyses will investigate meditational role of substance use and cognitive outcomes risk for addiction.

2.17 What is the proposed frequency of analyses?

Interim analyses at 12 and 24-month follow up will be conducted to confirm that the trial has had expected effects on drinking and illicit drug use behaviour and to check that there are no harmful effects of the intervention. These findings will be conducted by the data management committee and reported to the independent data monitoring committee who will keep results confidential unless harmful effects are indicated and recommendations for trial termination are made. Cognitive and addiction outcomes will be analysed at the end of the trial.

2.18 Are there any planned subgroup analyses?

Secondary analyses will be conducted to examine population-level effects of the intervention on substance-related behaviors and related cognitive outcomes[29]. Analyses described above will repeated on the low risk sample.

2.19 Has any pilot study been carried out using this design?

Three previous randomized trials (one cluster RCT) have been conducted using this same design, with the Adventure Trial most matching the proposed study [28, 29]. The PI is currently conducting qualitative interviews and focus groups with high school students in Montreal, to gather information necessary for the French adaptation of the Preventure Programme. This work will be completed by June, 2011.The PI also successfully completed a randomized trial of personality-targeted interventions for a primarily French-speaking adult substance abusing population [94] in Montreal, and has used the screening tool with Québéquois youth [22], so there is good reason to expect that the French adaptation of this programme will produce similar effects with Montreal students as it has with high school students in British Columbia, Nova Scotia [27] and London, UK [25, 26].

3. Trial Management

3.1 What are the arrangements for day to day management of the trial?

A trial management committee will consist of the study Principal Investigators (PIs) and named collaborators. Monthly meetings will take place between PIs and the trial coordinator and clinician. A full-time trial coordinator

will be responsible for ethical aspects of the study, school recruitment, scheduling follow-up assessments and communication. The trial coordinator will be blind to school allocation. The trial coordinator will be expected to publish the trial protocol in Year 2. A full-time clinical post-doctoral researcher will be responsible for all clinical aspects of this trial, including intervention translation, training, teacher supervision, evaluation of treatment integrity, and knowledge transfer and dissemination (training workshops and published therapy manuals). This person will be the only person aware of school allocation to intervention. A full-time research assistant and two PhD students will be employed to assist with data collection, data management, and study write up. They will be blind to treatment allocation. All data entry will be done directly into the web-based assessment platform and separate case report forms (school and participant) will log participant consent, contact information (at school and at home) and whether follow-up was completed. See Budget Justification for more detail on trial personnel.

3.2 What will be the role of each principal applicant and co-applicant proposed?

Dr. Patricia Conrod, Prof. Agrégé, CHU Ste Justine, UdeM, will be Principal Investigator (PI) on this trial and has expertise in school-based personality interventions and clinical trials with adolescents. She will oversee modification of interventions to French format, training of clinical and research staff, overall trial progress, data reporting, quality control of data, financial aspects and ethical issues. Dr. Robert Pihl, Professor of Psychology, McGill University, has extensive experience in alcohol research, will co-surpervise PhD Students with the PI in the McGill Clinical Psychology Programme or the Sciences Biomédicales programme at UdM, providing the trial PhD students with excellent clinical and academic training, as well as undergraduate trainees who will assist with data acquisition. Dr. Jordan Peterson, Professeur of Psychology, UofT, has a strong record in computerised assessment of personality, general cognition and executive functions. He will oversee maintenance of the data acquisition platform. Dr. Jean Seguin, Prof. Agrégé, UdM will provide input on French translation of interventions, and the assessment battery. He has extensive experience with neuropsychological assessment of adolescents in Quebec. He will also supervise clinical PhD students and undergraduate students in psychology, who by assisting with data acquisition will gain valuable experience in clinical research. Dr. Benoit Masse is based at Unité de recherché Clinique appliquée (URCA), at CHU-Ste-Justine and Associate Professor at UdeM and is an experienced biostatistician with strengths in quantitative methods and clinical trials designs. He will oversee all the statistical aspects of this trial, including data monitoring and analyses. M. Tazi and Mdm. Couillard are Directors of large French-speaking secondary schools in Montreal who will contribute expertise on implementation of preventative programmes in schools and assessment of academic outcomes and are currently collaborating on the French adaptation of the Preventure Programme. Mme Sophie Desjardins, drug and alcohol prevention, Commission scolaire de Montréal (CSDM), will provide assistance with ethic review and recruitment of schools within the CSDM, as will Viviane Brian, Consultant in Health Promotion and Prevention for the Lester B. Pearson School Board. Dr. Nancy Haley, M.D., Paediatrician, Montreal Public Health Department will assist with knowledge transfer activities. Maeve O'Leary-Barrett, collaborator and McGill Clinical Psychology PhD student was the trial coordinator of the Adventure Trial in London [28] and will provide input on study design and school recruitment.

3.3 Describe the trial steering committee and if relevant the data safety and monitoring committee.

An executive committee will consist of the Study Chair (Conrod) and the co-investigators of the trial. A Steering Committee will consist of the executive committee and all named collaborators. A scientific advisory committee will provide guidance on a range of scientific and clinical issues related to this protocol. A Data Management Committee (DMC), with Dr. Masse as executive, will oversee randomisation of schools (including matching schools on important demographics), data management, conduct interim analyses of trial effects on substance related behaviour (12 month outcomes and 24 months), as well as data analyses at the end of the study. This committee will also ensure that research staff are appropriately blind to randomisation. An independent Data and Safety Monitoring Committee (DSMC) will consist of experts not involved in the planning and the conduct of this study and free from conflicts of interest or obligations to the investigator. Members will be appointed by the PI and the DMC executive. See Appendix I for terms of reference of all committees

STATISTICAL ANALYSIS PLAN FOR CO-VENTURE TRIAL

A Cluster Randomized Trial Investigating the Effects of Selective Intervention on Adolescent Cognitive Development and Addiction

Study Title: A cluster-randomized controlled trial evaluating the effects of delaying onset of adolescent substance abuse on cognitive development and addiction following a selective, personality-targeted intervention programme: the Co-Venture trial.

French Title: Un essai randomisé par grappe évaluant l'effet de retarder l'âge d'abus de substances chez les adolescents sur le développement cognitif et sur la dépendance grâce à l'intervention visant les facteurs de risque personnels: Essai clinique Co-venture

CIHR grant ID: funding reference number MOP 114887
NCT Number: NCT01655615

Analysis plan authors Patricia Conrod, Principal Investigator
Benoît Mâsse, Statistician and Co-Investigator
Golsa Dehghan, Statistician
Aram Mahmoud, Project coordinator
Gabrielle Grégoire, Statistician

Version 1.1

Date April 15, 2021

TABLE OF CONTENTS

TABLE OF CONTENTS 2
Abbreviations 5
SECTION 1: ADMINISTRATIVE INFORMATION 6
1. Title and trial registration 6
1.a Title 6
1.b Trial registration 6
2. Statistical Analysis Plan version 6
3. Protocol version 6
4. Description of this document 6
4. Statistical Analysis Plan revisions 7
5. Roles and responsibilities 7
SECTION 2: INTRODUCTION 8
6. Background 8
7. Study Objectives 9
SECTION 3: TRIAL METHODS 9
8. Trial design 9
9. Randomisation 10
10. Sample size calculation for the primary outcome 10
11. Framework 11
12. Statistical interim analyses and stopping guidance 11
13. Timing of final analysis 11
14. Visit map: Outcomes and main interest variables collection time points 11
15. Confidence intervals and p-values 13
16. Description of any planned adjustments for multiplicity, and if so, including how the type 1 error is to be controlled 13
18. Compliance/or adherence and protocols deviations 13
18.a How adherence to the intervention will be presented 13
18.b Fidelity of interventions 15

18.c Description of protocol deviation for the trial 16
18.d Description of which protocol deviation will be summarized 16
19. Definition of Analysis populations e.g. modified ITT (mITT), complete case 17
20. Reporting of screening data to describe representativeness of trial sample....................17
21. Summary of eligibility criteria 19
22. Information to be included in the CONSORT flow diagram 19
23. Withdrawal/Follow-up-level of withdrawal 19
23.a Level of school withdrawal 21
23.b Reasons and details of school children withdrawal....................22
24. Baseline School and Students characteristics and descriptive statistics 22
24.a List of baseline characteristics 22
24.b Details on how baseline characteristics will be descriptively summarized 22
25. Outcomes 22
25.a Definition; Description and timing; Specific measurements and units; Any calculation or transformation; Used to derive an outcome 22
25.b Primary outcome 23
25.c Secondary outcomes....................23
26. Analysis methods 23
26.a Primary analysis methods used for the primary outcome....................23
26.b Supplementary analysis methods used for the primary outcome 23
26.c Analysis methods used for the secondary outcomes 23
26.b Adjustments for primary and secondary outcomes 24
26.c Planned sensitivity analyses....................24
26.c Planned subgroup analyses....................25
27. Missing data 25
27.a Accounting for Attrition 25
27.a Reporting and assumptions/statistical methods to handle missing data 25
28. Other analysis....................26
29. Harms 26

30. Statistical Software....26
31. References....27
31.a Reference list....27
31.b Reference to Data Management Plan....34
31.c Reference to Trial master file and statistical master file....34
31.d Reference to other Standardized operating procedures or documents....34
APPENDIX....35
Appendix 1: Timing of outcome assessments....35
Appendix 2: CONSORT diagram for the final study sample (Co-venture flow diagram)....36
Appendix 3: Additional information on Outcome measures. 37

Abbreviations

ACRU	Applied Clinical Research Unit
AS	Anxiety sensitivity
BSI	Brief Symptom Inventory
CATS	Children's Automatic Thoughts Scale
CFT	Cultures Figure Task
CMQ	Cannabis Motives Questionnaire
CTS-R	The Cognitive Therapy Scale-Revised
CMS	Child Memory Scales
DEP-ADO	Detection of alcohol and drug problems in adolescents questionnaire
DMQ	Drinking Motives Questionnaire
IMP	Impulsivity
NT	Negative thinking
MITI 3.0	Motivational Interviewing Treatment Integrity
PALP	Passive Avoidance Learning Paradigm
SDQ	Strengths & Difficulties Questionnaires
PIFA	Preventure Intervention Fidelity and Adherence
SS	Sensation seeking
SUD	Substance use disorder
SURPS	Substance Use Risk Profile Scale
SWM	Spatial working memory task

SECTION 1: ADMINISTRATIVE INFORMATION

1. Title and trial registration

1.a Title

Title: Statistical analysis plan for Co-venture Trial: A Cluster Randomized Trial Investigating the Effects of Selective Intervention on Adolescent Cognitive Development and Addiction

Sub-Title: Statistical analysis plan for Co-venture Trial

1.b Trial registration

Trial registration number: NCT01655615

2. Statistical Analysis Plan version

Version 1.1 Date: April 15, 2021

3. Protocol version

This document has been written based on information contained in study protocol Version 2.0 October 31th, 2016 (Version 1.0 August 2, 2012)

4. Description of this document

This document describes statistical analysis plan (SAP) for Co-Venture trial. The SAP was developed according to guidelines found in ICH E9[1]. This SAP contains more technical and detailed elaboration of principal features of the analysis describes in the protocol[1], and includes procedures for executing the statistical analysis of the primary and main secondary variables. Exploratory analyses not identified in the SAP will be clearly identified in the reporting as well as any deviation in the analyses from this original SAP.

The SAP should be executed once the trial database lock (DBL) has been achieved. Analyses will not be blind for the randomization group. All the analyses will strictly follow the Statistical Analysis Plan (SAP) described in this document. Analyses will be done under the responsibility of the trial statistician at the Data Coordinating Center (Applied Clinical Research Unit (ACRU), Hôpital Sainte-Justine, Montreal). The study Principal Investigator

[1] International Conference on Harmonisation of Technical Requirements for Registration of Pharmaceuticals for Human Use. ICH Harmonised Tripartite Guideline: Statistical Principles for Clinical Trials E9. London, England: European Medicines Agency; 1998.

and the Steering Committee will be kept blind to the randomization group until final disclosure of the study results.

4. Statistical Analysis Plan revisions

Protocol version	Updated SAP version	Section number	Description of and reason for change	Date changed
2.0	1.1			

5. Roles and responsibilities

Patricia Conrod, Principal Investigator
Robert Pihl, Co-Investigator
Sherry Stewart, Co-Investigator
Jean Séguin, Co-Investigator
Benoît Mâsse, Co- Investigator and Statistician

Authorized and approved by Trial steering and management Committee

	Signature	Date
Patricia Conrod	…… .	………May 5, 2021
Robert Pihl	………………………………………………….	
Sherry Stewart	………………………………………………….	
Jean Séguin	………………………………………………….	

Benoît Mâsse .... May 5, 2021

SECTION 2: INTRODUCTION

6. Background

Adolescent substance use is associated with a myriad of immediate and long-term negative consequences [2], Onset of alcohol use at or before 14 years of age predicts an increased risk of developing alcohol and drug use disorders [3], [4], [5] and other mental health problems [6], [7], [8], poor academic performance [2], [9], high school and college drop-out [10], [11], risky sexual behaviors [12], [13] and injuries [14]. Hazardous drinking patterns such as binge drinking are on the rise among youth worldwide [15], [16]. Statistics in the Canadian province of Quebec are striking and suggest that Quebec youth may initiate drinking earlier and show an accelerated growth in substance use relative to other North American youth. By grade 8, 44.4% of Quebec students have consumed alcohol, compared to the Canadian national average of less than 20% [17] and 29.3% in the USA [18]. By the end of high school, 76.5% of Quebec students report binge drinking and about 10% screen positive for a substance use disorder[39].

Adolescent brains may be particularly susceptible to damage from alcohol use due to the significant neuro-maturation occurring throughout this period [2]. This has been shown to be true in rodent models, where alcohol-neurodegeneration is more severe in adolescent than adult brains [19]. Enhanced susceptibility to alcohol-induced neurodegeneration has also been observed in human adolescents [20].

Alcoholism in adulthood has been associated with cognitive impairments across multiple domains [21],[22], [23]. Moderate deficits have also been reported in adolescents with less chronic alcohol use disorders, including impairments in verbal and non-verbal memory, attention, executive functions, and visuospatial performance [24], [25], [26]. In addition, cognitive deficits have been observed in the non-problematic, social drinking population [27], with the suggestion that there is a continuum of deficits related to quantity of alcohol consumption [28]. Because the majority of neuropsychological studies with adolescents are cross-sectional, it has been difficult to conclude whether the cognitive abnormalities described are causal or consequential to alcohol misuse. There is a large literature indicating that two major risk factors for adolescent-onset alcohol misuse, namely family history of alcoholism and adolescent onset psychopathology [29], are associated with brain abnormalities that are also seen in youth with other risk factors for substance use (e.g., externalising personality or psychopathology) [30], [31], [32], [33], [34]. There are significant discrepancies across studies examining the prospective associations between cognitive functioning and substance use [25], [35], [36] suggesting that the association between these variables merits further investigation.

Current theories of adolescent brain development propose that a delay in the development of cognitive functioning (sometimes referred to by some as a 'maturational gap'[37]), as opposed to stable cognitive deficits, may account for the increased risk taking and attentional difficulties seen in adolescence, as well as individual differences between youth[31]. Despite the existence of several longitudinal studies on this topic (e.g., IMAGEN; [38]), causality remains difficult to establish because lower (or delayed growth in) cognitive functioning might predict early onset substance use, but exposure to substances might further exacerbate cognitive development, particularly in the most vulnerable youth. It is thus important to assess substance use and cognitive functioning at multiple time points in a longitudinal design to examine the developmental sequence and interactions between the uptake and growth in alcohol consumption and cognitive development. In addition, cognitive functioning should be assessed prior to alcohol use onset.

7. Study Objectives

As a primary outcome, this longitudinal cluster-randomized controlled trial will examine the impact of Preventure on reducing addiction risk in adolescents over a 5-year period.

The primary research hypothesis is that fewer high-risk youth will meet criteria for a substance use disorder 4 years post-intervention than high-risk youth in intervention relative to control schools.

The secondary intermediate objectives are to determine whether the short-term impact of the intervention on delaying substance use onset at one year post-intervention, and intervention effects will improve cognitive outcome, as measured by neuropsychological tasks.

Secondary outcomes will examine the intervention effects on mental health outcomes 4 years post-intervention, as measured by self-report mental health screening measures.

SECTION 3: TRIAL METHODS

8. Trial design

This study is a cluster-randomized controlled trial designed to test the efficacy of Preventure personality-targeted interventions on substance use, cognitive and mental health outcomes in selected, high-risk adolescents attending school in the greater Montreal Area (Quebec, Canada).

9. Randomisation

A dynamic allocation (DA) method will be used to allocate schools to intervention or control arms to balance baseline prognostic factors at the cluster (school) level. The Data Coordinating Centre statistician at Hôpital Sainte-Justine (Benoît Mâsse, PI) generates randomization lists for school. The randomization process consists of computer-generated random lists of intervention allocations to school according to the Pocock–Simon modified minimization method to ensure balance across two covariates, the language of school(French, English) and the gender with three categories describing the proportion of boys in the school (< 25% boys, 25-75% boys, ≥ 75% boys). Details of the randomisation method are held securely at the Data Coordinating center (ACRU).

10. Sample size calculation for the primary outcome

Sample size calculations are conducted according to the sample needed for the primary long-term analyses, namely the onset of substance use disorders 4-years post-intervention in the sub-group of students identified at high-risk in grade 7. As the school (cluster) is the unit of randomisation, the sample size determination accounts for the clustering of students within schools. According to data from a local epidemiologic study [39] and a previous personality-targeted intervention trial[40], the expected proportion of students with a substance use disorder in grade 11 in the control group is 10%. The minimum detectable reduction is set to 50% (i.e., 10% substance use disorders in grade 11 in the control group versus 5% in the intervention group). Assuming that this proportion per school is approximately normally distributed (i.e., 95% of the schools would fall within two standard deviations of the mean, 10%, 7 %), we obtain a between-cluster coefficient of variation, k, of 0.35. In addition, we expect 50 students per school (40-45% of the full sample) to be identified as high-risk (average cluster size of 50), following previous personality-targeted intervention studies [414243]. With these assumptions (using formula 7.7, [44]), the number of schools (clusters) per arm needed to detect a 50% reduction in the proportion of drinkers (i.e., from 10% to 5%) with 80% power and a false positive error rate of 2.5% is 15, giving a total of 30 schools or approximately 1500 high-risk students. We plan to recruit two additional schools (30+ 2 = 32 schools total) to account for potential attrition at the school-level.

For the secondary outcomes (other measures of substance use, mental health and cognition), we will use the observed effect sizes from the IMAGEN study[38] on 2000 14 year-old European participants who completed neuropsychological assessments. Binge drinkers showed reduced IQ and a greater reward bias on the Passive Avoidance Learning Paradigm [45] relative to non-bingers where observed effect sizes (d) for these mean differences ranged

from 0.23-0.25[46]. Larger mean differences are expected in our study since our follow-up is longer. We also expect more cognitive development to take place in intervention participants as we expect reductions in substance use following the intervention to promote their cognitive development. Thus, we expect differences in cognitive development to increase over time between intervention and control groups. A sample size of 15 clusters per group with an average of 50 high-risk adolescents per cluster will achieve at least 90% power to detect a difference of at least 3.0 between the group means of cognitive functioning with a standard deviation of 15.0 (an effect size of 0.20) [47] and an intra-cluster correlation of 0.40 using a false positive error rate of 2.5%.

11. Framework

The objective of the trial is to test the superiority of the intervention from control on primary and secondary outcomes.

12. Statistical interim analyses and stopping guidance

Interim analyses at 12 and 24-month follow up will be conducted to confirm that the trial has had expected effects on drinking and illicit drug use behaviour and to check that there are no harmful effects of the intervention. These findings will be conducted by the data management committee and reported to the independent data monitoring committee who will keep results confidential unless harmful effects are indicated and recommendations for trial termination are made. Cognitive and addiction outcomes will be analysed at the end of the trial.

The executive and data monitoring committee decided that interim analyses would not be conducted to reduce any potential bias of introduced by interim findings on primary outcomes. As there wasn't any concern associated with potential harm from the intervention, in association with previous trials, there was no justification for introducing this potential bias to the study design.

13. Timing of final analysis

The final analysis of the intervention is conducted when all students have completed outcomes assessment at 48-months follow-up (post intervention).

14. Visit map: Outcomes and main interest variables collection time points

Outcomes and timing are presented in Visit Map Table 1 in details. Table 1 presents the outcomes and other variable that were collected at different time points. The length between follow-ups in each school is 12-month.

Table 1. Schedule of enrolment, interventions, and assessments of the study in schools.

		Pre-Intervention		Post-Intervention Number of months after the initiation of the intervention				
**Categories**	**Variables**	**Baseline**	**Intervention period**	**12-month**	**24-month**	**36-month**	**48-month**	**Variable type**
**Primary outcomes**	Substance use disorder	●		●	●	●	●	Continuous
**Secondary substance use**	Non-verbal functioning and abstract reasoning	●		●	●	●	●	Continuous
	Verbal and visual memory	●		●	●	●	●	Continuous
	Cognitive control and response inhibition	●		●	●	●	●	Continuous
	Spatial working memory	●		●	●	●	●	Continuous
	Mental health symptoms	●		●	●	●	●	Continuous
	Onset, Frequency and quantity of alcohol and drug use	●		●	●	●	●	Continuous
**Demographics**	Sex	●		●	●	●	●	Binary
	Socioeconomic status	●		●	●	●	●	Continuous
	Immigrant background	●		●	●	●	●	Categorical

Abbreviation:
*: Measured at school level.

15. Confidence intervals and p-values

All applicable statistical tests will be two-sided and at the 5% significance level, unless otherwise specified, with two-sided 95% confidence intervals presented whenever possible.

16. Description of any planned adjustments for multiplicity, and if so, including how the type 1 error is to be controlled

As there is a single primary endpoint, and the secondary endpoints are to be used only in providing additional supportive exploratory information, and no interim analysis on the primary outcome were done, there will be no adjustments for multiple testing.

18. Compliance/or adherence and protocols deviations

18.a How adherence to the intervention will be presented

Compliance will be defined as being able to conduct all five assessments and, for intervention schools, being able to provide interventions to 85% of all high-risk youth in their school. Based on previous studies, schools generally had little difficulty complying and implementing the protocol, with only one school dropping out of the Adventure Trial due to systematic problems within the school and an impending school closure. Another intervention school was unable to complete the protocol for running sufficient interventions sessions with HR students and was thus not included in the trial. However, the main objective of the Adventure Trial was to evaluate effectiveness of the Programme, so research staff in this previous study could not offer additional support to schools if they could not deliver a certain aspect of the intervention protocol. Effectiveness will not be evaluated in the proposed study, and the current protocol will allow research staff to assist schools in meeting demands of the research or intervention protocol, thus further improving compliance rates in the proposed trial. Based on recruitment rates from the three previous trials, student participation rates are anticipated as being >85%, and with a passive parental consent procedure, we should see very little attrition due to non-consent. One of the main sources of attrition in school-based research is requiring that parents provide active, signed consent for their child to participate. Passive consent was used at various stages in the three previous trials conducted by the PI and are easily justified to an ethics committee by our data suggesting that high risk youth are more likely to volunteer to participate in these preventative interventions, but less likely to be able to provide signed consent forms from their parents, likely due to personality and related cognitive difficulties in the child or the family.

The following table provides an estimate of degree of compliance. As does school-level follow-up rate at each year (see CONSORT Figure 2).

Table 2: Participation of high-risk youth in intervention sessions and format of intervention delivery by school.

**Intervention school**	**Intervention Delivery**	**High risk students**	**Students completed at least one intervention session**	**Students completed both intervention sessions**
1	School staff	38	23 (61%)	21 (55%)
2	Research Therapists	56	56 (100%)	55 (98%)
3	School Staff	54	54 (100%)	51 (94%)
4	Research Therapists	63	60 (95%)	44 (70%)
5	Research Therapists	56	35 (63%)	30 (54%)
6	School Staff	51	51 (100%)	51 (100%)
7	School Staff + Research Therapist	66	65 (98%)	59 (89%)
8	Research Therapists	52	49 (94%)	47 (90%)
9	School Staff + Research Therapist	57	29 (51%)	29 (51%)
10	School Staff + Research Therapist	86	68 (79%)	60 (70%)
11	School Staff	14	14 (100%)	13 (93%)
12	School Staff	21	17 (81%)	17 (81%)
13	School Staff	34	32 (94%)	31 (91%)
14	School Staff + Research Therapist	36	35 (97%)	28 (78%)
15	Research Therapist	25	25 (100%)	25 (100%)
		Total: 709	Mean: 86%	Mean: 79%

Training in intervention delivery will be offered to up to five staff-members per intervention school (e.g., school counsellors and special educational needs staff). Facilitators will attend a 3-day workshop reviewing the necessary therapeutic principles involving didactic instruction and role-play. Trainees will receive a minimum of 4 hours of supervision and feedback from the clinical team in conducting a full intervention with grade 8-10 students (not involved in the trial). High-risk youth in intervention schools will receive interventions from a trained school-based facilitator and co-facilitator. If schools do not have the resources to run all the intervention groups themselves, trained members of the clinical research team (master's

level therapists and a clinical psychology PhD student) will deliver some or all of the interventions (see Table 1 for details).

Fidelity and adherence were assessed on a random 30% of all intervention sessions, using the Preventure Intervention Fidelity and Adherence (PIFA). This scale was used developed by the principal investigator (PC) to evaluate adherence to 12 core treatment components of the Preventure program (e.g., goal setting, identifying and challenging automatic thoughts[48]). The scale also measures fidelity by rating quality of intervention delivery (see next section).

18.b Fidelity of interventions

A previously-described scale has been developed by the principal investigator (PC) to evaluate adherence to 12 core treatment components of the Preventure program (e.g., goal setting, identifying and challenging automatic thoughts [48]). The Cognitive Therapy Scale-Revised [68] and the Motivational Interviewing Treatment Integrity 3.0[49] will also be used to assess the quality of the therapy-specific skills demonstrated. Trainees will be given feedback on their performance based on these scales and each facilitator will be supported in attaining a sufficient level of program adherence and quality of delivery before running personality-targeted interventions with trial participants. The clinical research team using the three scales will evaluate a random 30% of all intervention sessions and each facilitator will be observed running at least one intervention session. In addition, an independent rater (MINT network trainer) will evaluate 10% of all intervention sessions. The evaluation data will be used to evaluate and validate the fidelity and quality of the interventions delivered.

Means fidelity/quality of supervision for each scale

Measure	Clinical Team
Fidelity Checklist (0 to6) [1]	3,2
Motivational Interviewing Scale (1 to 5) [2]	3,24
Cognitive Behavioral Scale (1 to 6) [3]	3,14

Means fidelity/quality of intervention for each scale

Measure	Clinical Team	Independent rater
Fidelity Checklist (0 to6) [1]	3,8	-
Motivational Interviewing Scale (1 to 5) [2]	3,3	3,7
Cognitive Therapy Scale (1 to 6) [3]	3,6	2,9

[1] A score of 3 is classified as ''satisfactory'' a score of 4 is classified as ''good'' to fidelity

[2] An average of 3.5 is beginning proficiency and average of 4 is competency

[3] A score between 3 and 4 is classified as ''competent'' to adherence

18.c Description of protocol deviation for the trial

In the end, some schools did require active parental consent for youth to participate in this trial; therefore, not all HR youth were recruited and were provided with interventions in the intervention condition. However, these schools will remain in the study, and considered “compliant”. Only schools that agreed and were able to facilitate follow-up every year were included in the study, regardless of follow-up rate (therefore, compliant). Schools that did not agree to continue to facilitate follow-up of students were considered dropout (non-compliant) and will not be included in **the intent to treat analysis**.

Protocol deviation are defined as departure from the procedures outlined in the protocol. We used a modified intention to treat (mITT) design including only schools that were randomized and followed during 5 years. At the individual level, mITT will be all high-risk students in the schools who consented to a trial and eligible to receive intervention (Regardless of whether or not they received it). Per-protocol analysis at the individual level will exclude the students who did not receive both intervention sessions.

18.d Description of which protocol deviation will be summarized

Protocol deviation (PD) will be classified prior to unblinding of intervention groups.

1. During the recruitment phase, five schools were recruited and drop out after the randomisation. Among those, three schools never launched screening and baseline survey, and so were not included. One school drop out after screening and one school did deliver intervention, but failed to comply with follow-up procedures, so was also dropped from the trial and not included in mITT.
2. There were some deviations related to the data collection and the questionnaire administration. In the DEP-ADO, a missing item (“last 30 days alcohol use”) was not assessed. Therefore, the positive screen was adjusted to “greater or equal to 18” (Red). Truancy is self-reported. The CMS had to be modified to increase ceiling effects (one additional item was added in year 3, 4 and 5). Finally, the academic records were not obtained.
3. Visit completed outside of the planned window since there were two waves of recruitment, students were recruited in the 7th grade in 2012 and 2013.
4. There were also some procedures not performed. We changed intervention adherence criterion. Overall 88% of HR students received intervention, but some schools had poor implementation, so we will control for adherence, rather than require a certain level of implementation). One school closed and half the students could not be followed individually, but school remains in the study, because they provided access to student information so that they could be followed individually. One school missed a follow-up survey in year 4 and instead completed two surveys 7 months apart in the final year: they are considered compliant and in the ITT.

19. Definition of Analysis populations e.g. modified ITT (mITT)

The mITT population is defined as all the schools (excluding schools dropped out after randomization, c.f. Figure 1, whose participation rate was too low along with administration difficulties).

The units of analysis are at both the student's level and school's level for all outcomes.

Schools included in mITT:

1. Completed baseline survey
2. Agreed to continue with study protocol and remain in study post baseline (Delivered 50% of follow-up surveys sessions 2/4)

Child included in mITT:

1. Assented and parent passive or active consent
2. Completed baseline survey
3. Provided reliable substance use (Dep-Ado, all 36 items used to identify SUD) and SURPS (All 23 items) data at baseline and year 5.
4. High Risk status participants (n=709) will be included.

Per-protocol will exclude participants that did not attend the two sessions of intervention.

20. Reporting of screening data to describe representativeness of trial sample

Co-Venture cohort, which includes 76% of all grade 7 students attending 31 secondary schools in the greater Montreal area (Quebec, Canada). This cohort represents 15% of all schools in the area and each of their respective school districts in size and deprivation indexes within 1.5 standard deviation.

Quality control is conducted using both automated algorithm-based functions and manual scanning of data. The DMC provides coded databases to study research assistants and PHD students responsible for data quality control and will oversee all quality control protocols.

We have developed a successful automated system for data quality control which included sham questions (Conrod et al., 2010), questions on participant motivation (Seguin, et al., (2009) and testing context (Schumann, et al., 2010) to assess reliability of information. The structure of the questionnaire, the repeat questions/sham questions and the testing context allow for significant confidence interval in the validity of the self-reported data.

Quality control flag applied:

On the primary outcome

- For the DEP-ADO, those who answered positively to the fake drug item (DEPADO Foil) were flagged and 0.4% of DEP-ADO Drug records were rejected.
- Secondly, those who report uncommon substance use (sum of (TRANQ, STEROIDS, SPEED, PCP, LSD, HEROIN, ECSTASY)>2.5) were also flagged and 0.1% of records were rejected.
- The SURPS results should indicate a minimum of variation. In the absence of variation in the answers, results were flagged and 0.4% of records were rejected)

On the secondary outcomes:

- CFT - There seems to be a significant year to year learning effect in this task (0.1% of CFT records rejected)
- The PALP's "worse than chance" QC flag identifies and excludes those with more than 30 commissions or with more than 30 omissions. With that flag, 17.6% of records were rejected.
- Just like the SURPS, if there was no variation in the SDQ answers, answers were rejected and 0.6% of records were rejected).
- For the CMS task, the time duration between the first part and the recall should not be negative, or longer than the time allotted in a period of class

A trial management committee will consist of the study Principal Investigators (PIs) and named collaborators. Monthly meetings will take place between PIs and the trial coordinator and clinician. A full-time trial coordinator will be responsible for ethical aspects of the study, school recruitment, scheduling follow-up assessments and communication. The trial coordinator will be blind to school allocation. The trial coordinator will be expected to publish the trial protocol in Year 2. A full-time clinical post-doctoral researcher will be responsible for all clinical aspects of this trial, including intervention translation, training, teacher supervision, evaluation of treatment integrity, and knowledge transfer and dissemination (training workshops and published therapy manuals). This person will be the only person aware of school allocation to intervention. A full-time research assistant and two PhD students will be employed to assist with data collection, data management, and study write up. They will be blind to treatment allocation. All data entry will be done directly into the web-based assessment platform and separate case report forms (school and participant) will log participant consent, contact information (at school and at home) and whether follow-up was completed.

21. Summary of eligibility criteria

Ages Eligible for Study: 12 Years to 17 Years (Child)
Sexes Eligible for Study: All
Accepts Healthy Volunteers: Yes

Selection of Schools:
Inclusion criteria: schools (public or private) able to recruit and willing to conduct at least one follow-up survey**.**

Exclusion criteria: Schools cannot be classified as having a majority of their students coded as special needs students, because these schools are smaller and the intervention protocol would have to be tailored for their particular needs.

Selection of high-risk youth:
Inclusion criteria: Students are that they be enrolled in Grade 7 and that they have provided active assent and passive parental consent to participate in the longitudinal survey and randomised trial phases of this study. Participants who score 1 standard deviation above the school mean on one of four subscales of the Substance Use Risk Profile Scale (SURPS) [50] will be selected to participate in the intervention groups, but all students will be followed on outcomes.

Exclusion criteria: There are no other exclusion criteria for participants.

22. Information to be included in the CONSORT flow diagram

The figure 2 , in Appendix 2, reports the flow of schools and students through the trial in accordance with the Consolidated Standards of Reporting Trials (CONSORT) extension statement for cluster trials [51]. The flow diagram includes the number of eligible and recruited schools, number of eligible and recruited students and then, by allocated group, the number of students who continued through the trial, the number of withdrawing at each time point, the number of lost to follow-up at each time point and the numbers included in the analysis.

23. Withdrawal/Follow-up-level of withdrawal

A REB- and school board-approved passive informed consent procedure involves asking parents to contact the school or research team if they do not wish their child to participate in the study. All assenting students will provide active written consent to participate in the study. An additional active parental written consent form will be provided to nine schools who request this procedure in addition to active youth consent.

School students who discontinue completing the data collection prior to the end of trial period were considered as missing data for time points that they missed but their baseline and following present data were kept in the dataset.

23.a Level of school withdrawal

Figure 1: Recruitment of schools and dropouts prior to finalization of school sample

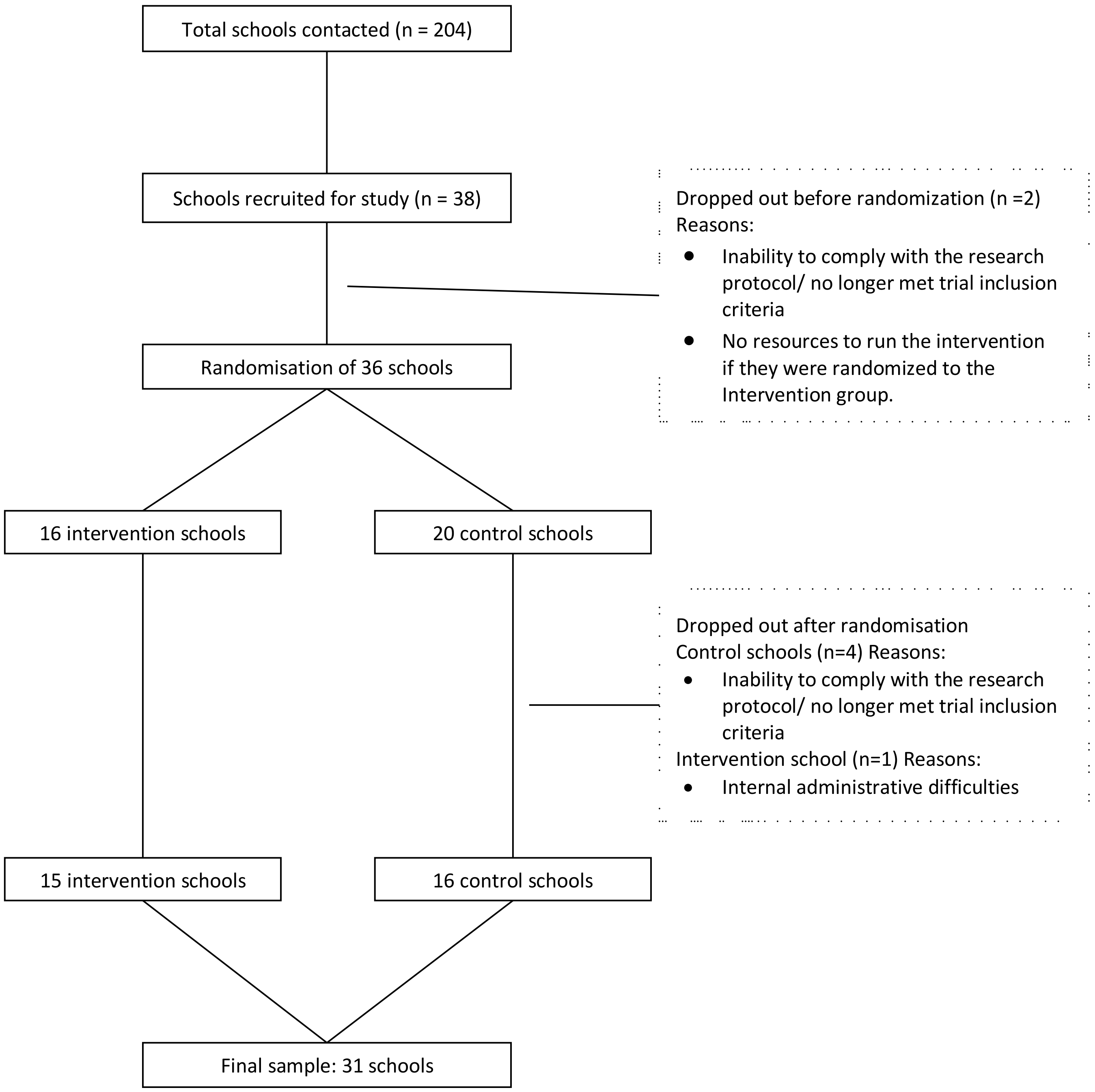

23.b Reasons and details of school children withdrawal

Most students drop out of a trial due to graduating from high school and moving to other schools, but this will not be a problem in this trial, as we do not expect to follow-up students beyond Grade 11. At the student level, the principal reasons of withdrawal are having left school (e.g. moving, conduct problems, academic difficulty). There are few cases of lost students because a school closed and one reported of death in the control condition.

24. Baseline School and Students characteristics and descriptive statistics

Baseline characteristics, collected at the time of commencing the trial, will be cross-tabulated according to the randomised group to check for appropriate balance and to provide an overview of the study population, both at the school and individual levels. Visual inspections will be provided to show prevalence/or average of primary/or secondary outcomes within each cluster level (i.e. school level) at each time point over the 5-year trial.

24.a List of baseline characteristics

There are summarized in dummy Tables.

24.b Details on how baseline characteristics will be descriptively summarized

At the school level, characteristics will include primary and secondary outcomes. At the individual level, variables include demographic variables as well as primary and secondary outcomes. The baseline characteristics of each group will be summarized as the mean, median, standard deviation, quartiles and range (min, max) for continuous variables; frequencies and percentages of individual/schools in each category for categorical variables.

It is expected that individual in the both collected allocated groups will, on average, be similar, given the randomization procedure.

Test of statistical significance will not be systematically done. Only descriptive data, as described above, will be presented. If substantial imbalance between randomization groups is identified in terms of any relevant variables not already being adjusted for in the primary analysis, additional adjusted sensitivity analyses may be performed.

25. Outcomes

25.a Definition; Description and timing; Specific measurements and units; Any calculation or transformation; Used to derive an outcome

See variable directory and Tables for description and timing.

25.b Primary outcome

Further details on assessment are provided in protocol and Appendix 3.

25.c Secondary outcomes

Major secondary outcomes have been defined in Appendix 3.

26. Analysis methods

26.a Primary analysis methods used for the primary outcome

The primary analyses will be the estimation of the effect of the intervention on substance use disorders in high-risk youth in intervention conditions relative to their control counterparts, at the year 5 follow-up (48 months post-intervention). The odds ratio (OR) will be used as the measure of intervention effect for the binary substance use disorders outcome. To account for within-cluster (school) variance, intervention effects will be examined using hierarchical generalized linear modelling (HGLM; using the SAS program, Proc GLIMMIX[52], [53]) using a Bernoulli distribution for the binary primary outcome. The HGLM model will be two-levels, with students as the first level, and nested within schools (clusters) as the second level of the model.

26.b Supplementary analysis methods used for the primary outcome

Similar to the primary analysis, supplementary analysis will done on the binary primary outcome (substance use disorders) over all post-intervention time points (12 - 48 months), to estimate the effect of the intervention on substance use disorders in high-risk youth in intervention conditions relative to their control counterparts over the 5-year trial (i.e. growth in substance use disorders). To account for within-cluster (school) variance, intervention effects will be examined using HGLM, and the outcomes will be centered at the 12-month follow-up to allow for comparisons between groups post-intervention. The model will be three-levels, with time points as the first level, nested within students as the second level, nested within schools as the third level of the model. When significant effects are revealed on growth, analyses will be conducted (i.e. HGLM) testing intervention effects on onset of use at specific time points while controlling for cluster, using tests based on the Huber-White sandwich estimate of variance.

Supplementary analysis will also be done on the estimation of the effect of the intervention on the continuous value of the primary outcome measure (DEP-ADO scores) between control and intervention group, at the year 5 follow-up (48 months post-intervention). Effect will be analyzed using a mixed effect linear model to compute adjusted mean difference between arms along with their standard errors. The model will be two-levels, with students as the first level, nested within schools as the second level of the model. If there is a violation of distribution assumption on outcomes, appropriate transformation will be used.

26.c Analysis methods used for the secondary outcomes

Using a similar method, secondary analyses will examine the 12-month intercept and the linear slopes (i.e. growth) from 12 - 48 months post-intervention between intervention and control conditions in alcohol and drug use behaviours. Growth terms will be used to detect prevention effects on growth in substance use behaviours over 4 years.

Similar analyses will be carried out for secondary and exploratory outcomes presented in Appendix 3.

26.b Adjustments for primary and secondary outcomes

To take into account the baseline prognostic factors at the cluster (school) level used for DA (c.f. Section Randomisation), all the results will be adjusted for language of the school, and the gender with three categories.

In case of imbalance for a variable at baseline, interaction and confounding effects will be tested according to its clinical significance.

Modification to protocol: Considering that immigration status has a very large protective effect on youth substance use (including in this sample), and that schools in Montreal vary significantly on this variable, we have modified the analysis plan to also include immigration status (immigrant, first generation, second generation) as a covariate.

26.c Planned sensitivity analyses

Sensitivity analyses will be implemented to examine plausibility of putative mechanisms underlying the effectiveness of the intervention as well as the interaction between intervention and participants' characteristics.

Sensitivity 1: Replace missing data for the binary primary outcome using multiple imputation (section 27.a).

Modification to protocol: The senior investigator never intended to report primary outcomes without replacing missing data. This was an oversight at the time of finalising the first analysis plan. We therefore have agreed to report primary outcomes after having replaced missing data using a full information method with 20 imputations (see 27.a). Therefore, this procedure will be followed for the primary and secondary analyses, and not only for Sensitivity analysis 1.

Sensitivity 2: Test effect in HR students who indeed received the intervention rather than mITT (ie Per Protocol analysis)

Sensitivity 3: Interaction between HR status and Intervention effect – does the intervention effect reveal itself at a population level?

26.c Planned subgroup analyses

Formal subgroup analyses were not planned in the protocol.

27. Missing data

27.a Accounting for Attrition

Each student will contribute annual observations until his/her end of follow-up (i.e. Grade 11). It is possible that attrition will be non-random or that some students may not show up for some of their annual assessments. To deal with such potential biases due to non-random attrition, individual annual observations will be re-weighed using the Inverse Probability of Weights (IPW)[54, 55, 56,] which will be estimated through HGLM model with a binary outcome (being present or absent at a given annual visit) based on the selected students baseline characteristics.

27.a Reporting and assumptions/statistical methods to handle missing data

As missing primary outcome values (DEP-ADO scores at 48 months post-intervention) might depend on observed data and/or missing data, and due to the large amount of missing data for the binary primary outcome at 48 months post-intervention (see CONSORT flow diagram, Appendix 2), primary analysis will be performed with imputation for missing data using Multiple Imputation (MI). Missing data will be imputed under Missing At Random (MAR) assumption, and odds ratios estimates for each imputed data set will be obtained. The imputation will be repeated 20 times and Rubin's rule will be used to combine estimates and standard errors [57].

This represents a change to the original analysis protocol, which did not acknowledge that the potential bias in only considering data that from participants who were available for follow-up. Outcomes are expected to be affected by risk status and substance use outcomes, therefore intent-to-treat analysis will include all participants. Furthermore, as missing data is also at random and highly dependent on participant simply being present on the day of testing at school, there are also cases of missing data at only one timepoint of the 5 assessments. Omitting participants on the basis of one missing timepoint (i.e., listwise) would lead to very large attrition and lost data that would otherwise be useful in the evaluation of the outcome (either through estimate final outcome, or by providing outcomes at other timepoints).

28. Other analysis

Exploratory analyses not identified in the SAP will be clearly identified in the reporting as well as any deviation in the analyses from this original SAP.

29. Harms

There is a risk to implementing interventions with youth, in that certain prevention programs have shown negative effects[58, 59,] including consistent negative effects in non-cognitive-behavioural counselling and social work interventions[60]. However, the proposed personality-targeted interventions incorporate therapeutic techniques associated with beneficial outcomes[61] and have been shown to have substantial positive effects in three separate populations across Canada[43] and the United Kingdom for individuals participating in interventions[62, 63, 40,] as well as indirect population-level benefits across the school grade[64].

There are possible risks in implementation of selective interventions by educational professionals in a school context, both for adolescent participants and facilitators. The targeted nature of the programme necessitates a careful introduction of the study and interventions to participants in order to avoid participants being labelled or stigmatised. All self-report data will be kept confidential and parents and teachers consent to not having access to their child's self-report information. Selection criteria are kept confidential and are only revealed to the staff members who are trained to deliver the programme and who undergo careful training on the importance of confidentiality around this programme. This programme works with educational professionals and school counsellors who are professionally trained to deal with issues of confidentiality, student distress and at risk cases. A clinical psychologist will be available to provide research staff and schools with additional clinical support should issues of risk be revealed. These concerns have been addressed and evaluated throughout the delivery the Adventure Trial, a teacher-delivered personality-targeted intervention trial[40], as well as during intervention delivery by psychologists[62, 43, 63,65]. The proposed protocol is designed to minimise any risk to those involved.

As such, we decided it was not necessary to perform interim data analyses.

30. Statistical Software

SAS Statistical Software, Version 9.4

31. References

31.a Reference list

1. O'Leary-Barrett M, Mâsse B, Pihl RO, Stewart SH, Séguin JR, Conrod PJ. A cluster-randomized controlled trial evaluating the effects of delaying onset of adolescent substance abuse on cognitive development and addiction following a selective, personality-targeted intervention programme: the Co-Venture trial. *Addiction*. 2017. doi:10.1111/add.13876

2. Zeigler D, Wang C, Yoast R, … BD-P, 2005 undefined. The neurocognitive effects of alcohol on adolescents and college students. *Elsevier*. https://www.sciencedirect.com/science/article/pii/S0091743504002658. Accessed July 30, 2019.

3. Grant B, abuse DD-J of substance, 1998 undefined. Age of onset of drug use and its association with DSM-IV drug abuse and dependence: results from the National Longitudinal Alcohol Epidemiologic Survey. *Elsevier*. https://www.sciencedirect.com/science/article/pii/S089932899980131X. Accessed July 30, 2019.

4. Grant BF, Dawson DA. AGE AT ONSET OF ALCOHOL USE AND ITS ASSOCIATION WITH DSM-IV ALCOHOL ABUSE AND DEPENDENCE: Results from the National Longitudinal Alcohol Epidemiologic Survey.

5. Newton-Howes G, Boden JM. Relation between age of first drinking and mental health and alcohol and drug disorders in adulthood: Evidence from a 35-year cohort study. *Addiction*. 2016. doi:10.1111/add.13230

6. Bott K, Meyer C, Rumpf H-J, Hapke U, John U. Psychiatric disorders among at-risk consumers of alcohol in the general population. *J Stud Alcohol*. 2015. doi:10.15288/jsa.2005.66.246

7. Langenbach T, Spönlein A, Overfeld E, et al. Axis I comorbidity in adolescent inpatients referred for treatment of substance use disorders. *Child Adolesc Psychiatry Ment Health*. 2010. doi:10.1186/1753-2000-4-25

8. Welsh J, Knight J, Hou S, … MM-J of A, 2017 undefined. Association between substance use diagnoses and psychiatric disorders in an adolescent and young adult clinic-based population. *Elsevier*. https://www.sciencedirect.com/science/article/pii/S1054139X16309648. Accessed July 30, 2019.

9. Wechsler H, Lee JE, Kuo M, Lee H. College binge drinking in the 1990s: A continuing problem results of the harvard school of public health 1999 college alcohol study. *J Am Coll Health Assoc*. 2000. doi:10.1080/07448480009599305

10. Patrick ME, Schulenberg JE, O'Malley PM. High School Substance Use as a Predictor of College Attendance, Completion, and Dropout: A National Multicohort Longitudinal Study. *Youth Soc*. 2016. doi:10.1177/0044118X13508961

11. Townsend L, Flisher AJ, King G. A systematic review of the relationship between high school dropout and substance use. *Clin Child Fam Psychol Rev*. 2007. doi:10.1007/s10567-007-0023-7

12. Halpern-Felsher B, Millstein S, Health JE-J of A, 1996 undefined. Relationship of alcohol use and risky sexual behavior: a review and analysis of findings. *Elsevier*. https://www.sciencedirect.com/science/article/pii/S1054139X96000249. Accessed July 30, 2019.

13. Tapert S, Aarons G, Sedlar G, Health SB-J of A, 2001 undefined. Adolescent substance use and sexual risk-taking behavior. *Elsevier*. https://www.sciencedirect.com/science/article/pii/S1054139X00001695. Accessed July 30, 2019.

14. care RP-P emergency, 2000 undefined. Alcohol and injury in adolescents. *journals.lww.com*. https://journals.lww.com/pec-online/fulltext/2000/10000/alcohol_and_injury_in_adolescents.3.aspx. Accessed July 30, 2019.

15. Substance WHOM of. *Global Status Report on Alcohol and Health, 2014*.; 2014. https://books.google.ca/books?hl=en&lr=&id=HbQXDAAAQBAJ&oi=fnd&pg=PR2&dq=World+Health+Organization,+Global+status+report+on+alcohol+and+health.+2014:+Geneva.&ots=PbtAEkS4yC&sig=kR7mlS8Cvlw-gupDtUNiTHF5AR0. Accessed July 30, 2019.

16. Lancet T. Calling time on young people's alcohol consumption. 2008.

17. Hammond, D., Ahmed, R., Yang, W.S., Brukhalter, R., and Leatherdale, S. Illicit substance use among Canadian youth: trends between 2002 and 2008. *Can J Public Heal*. 2011;102(1):7-12.

18. Johnston, L.D., O'Malley, P.M., Bachman, J.G., and Schulenberg JE. *Monitoring the Future National Results on Adolescent Drug Use: Overview of Key Findings, 2010*.; 2011.

19. Crews FT, Braun CJ, Hoplight B, Switzer RC, Knapp DJ. Binge ethanol consumption causes differential brain damage in young adolescent rats compared with adult rats. *Alcohol Clin Exp Res*. 2000. doi:10.1111/j.1530-0277.2000.tb01973.x

20. Nixon K, psychiatry JM-C opinion in, 2010 undefined. Adolescence as a critical window for developing an alcohol use disorder: current findings in neuroscience. *ncbi.nlm.nih.gov*. https://www.ncbi.nlm.nih.gov/pmc/articles/PMC3149806/. Accessed July 30, 2019.

21. Bühler M, Vollstädt-Klein S, Kobiella A, et al. Nicotine Dependence Is Characterized by Disordered Reward Processing in a Network Driving Motivation. 2010. doi:10.1016/j.biopsych.2009.10.029

22. Castellanos-Ryan N, Rubia K, Conrod PJ. Response Inhibition and Reward Response Bias Mediate the Predictive Relationships Between Impulsivity and Sensation Seeking and Common and Unique Variance in Conduct Disorder and Substance Misuse. *Alcohol Clin Exp Res*. 2011. doi:10.1111/j.1530-0277.2010.01331.x

23. Goldstein RZ, Parvaz MA, Maloney T, et al. Compromised sensitivity to monetary reward in current cocaine users: An ERP study. *Psychophysiology*. 2008. doi:10.1111/j.1469-8986.2008.00670.x

24. Tapert SF, Brown GG, Kindermann SS, Cheung EH, Frank LR, Brown SA. fMRI measurement of brain dysfunction in alcohol-dependent young women. *Alcohol Clin Exp Res*. 2001. doi:10.1111/j.1530-0277.2001.tb02204.x

25. Tapert SF, Brown SA. Substance dependence, family history of alcohol dependence and neuropsychological functioning adolescence. *Addiction*. 2000. doi:10.1046/j.1360-0443.2000.95710436.x

26. Brown SA, Tapert SF, Granholm E, Delis DC. Neurocognitive Functioning of Adolescents: Effects of Protracted Alcohol Use. *Alcohol Clin Exp Res*. 2000;24(2):164-171. doi:10.1111/j.1530-0277.2000.tb04586.x

27. Parsons OA. Neurocognitive Deficits in Alcoholics and Social Drinkers: A Continuum? *Alcohol Clin Exp Res*. 2006. doi:10.1111/j.1530-0277.1998.tb03895.x

28. Squeglia L, Spadoni A, … MI-P of, 2009 undefined. Initiating moderate to heavy alcohol use predicts changes in neuropsychological functioning for adolescent girls and boys. *psycnet.apa.org*. https://psycnet.apa.org/record/2009-24023-019. Accessed July 30, 2019.

29. Kirisci L, Tarter R, Reynolds M, Behaviors MV-A, 2006 undefined. Individual differences in childhood neurobehavior disinhibition predict decision to desist substance use during adolescence and substance use disorder in young. *Elsevier*. https://www.sciencedirect.com/science/article/pii/S0306460305001541. Accessed July 30, 2019.

30. Harden P, Psychology RP-J of A, 1995 undefined. Cognitive function, cardiovascular reactivity, and behavior in boys at high risk for alcoholism. *psycnet.apa.org*. https://psycnet.apa.org/record/1995-16853-001. Accessed July 30, 2019.

31. Logan GD, Sergeant JA. Oosterlaan J , Logan GD , Sergeant JA . Response inhibition in AD / HD , CD , comorbid AD / HD + CD , anxious , and control children : a meta-analysis of studies with the stop task . J Child Psyc ... Response Inhibition in AD \ HD , CD , Comorbid AD \ HD . 2015. doi:10.1111/1469-7610.00336

32. Peterson JB, Finn PR, Pihl RO. Cognitive dysfunction and the inherited predisposition to alcoholism. *J Stud Alcohol*. 2015. doi:10.15288/jsa.1992.53.154

33. Rubia K, Smith A, Brammer M, psychiatry ET-B, 2007 undefined. Temporal lobe dysfunction in medication-naive boys with attention-deficit/hyperactivity disorder during attention allocation and its relation to response variability. *Elsevier*. https://www.sciencedirect.com/science/article/pii/S0006322307001928. Accessed July 30, 2019.

34. Sonuga-Barke EJS, Dalen L, Daley D, Remington B. Are planning, working memory, and inhibition associated with individual differences in preschool ADHD symptoms? *Dev Neuropsychol*. 2002. doi:10.1207/S15326942DN2103_3

35. Castellanos-Ryan N, Seguin J, … FV-TBJ of, 2013 undefined. Impact of a 2-year multimodal intervention for disruptive 6-year-olds on substance use in adolescence: randomised controlled trial. *cambridge.org*. https://www.cambridge.org/core/journals/the-british-journal-of-psychiatry/article/impact-of-a-2year-multimodal-intervention-for-disruptive-6yearolds-on-substance-use-in-adolescence-randomised-controlled-trial/7A95443B922E775AA3CC4ABFE39610E4. Accessed July 30, 2019.

36. Castellanos-Ryan N, Struve M, Whelan R, et al. Neural and cognitive correlates of the common and specific variance across externalizing problems in young adolescence. *Am J Psychiatry*. 2014. doi:10.1176/appi.ajp.2014.13111499

37. review LS-D, 2008 undefined. A social neuroscience perspective on adolescent risk-taking. *Elsevier*. https://www.sciencedirect.com/science/article/pii/S0273229707000536. Accessed July 30, 2019.

38. Schumann G, Loth E, Banaschewski T, … AB-M, 2010 undefined. The IMAGEN study: reinforcement-related behaviour in normal brain function and psychopathology. *nature.com*. https://www.nature.com/articles/mp20104. Accessed July 30, 2019.

39. Dubé, G., Bordeleau, M., Cazale, L., Fournier, C., Traoré, I., Plante, N. et al. [Alcohol and drug use], in [Quebec survey of tobacco, alcohol and drug use, and gambling in high school students, 2008], Dubé G and others, Editors. *[Quebec Quebec Institute of Statistics]: Quebec]*. 2009:83-124.

40. O'Leary-Barrett M, … CM-J of the A, 2010 undefined. Personality-targeted interventions delay uptake of drinking and decrease risk of alcohol-related problems when delivered by teachers. *Elsevier*. https://www.sciencedirect.com/science/article/pii/S0890856710004521. Accessed July 30, 2019.

41. Conrod P, Castellanos Ryan N, Mackie CJ. Long-Term Effects of a Personality-Targeted Intervention to Reduce Alcohol Use in Adolescents Preventure View project Inter-Venture Trial View project. *Artic J Consult Clin Psychol*. 2011. doi:10.1037/a0022997

42. Conrod P, O'Leary-Barrett M, … NN-J, 2013 undefined. Effectiveness of a selective, personality-targeted prevention program for adolescent alcohol use and misuse: a cluster randomized controlled trial. *jamanetwork.com*. https://jamanetwork.com/journals/jamapsychiatry/article-abstract/1558064. Accessed July 30, 2019.

43. Conrod PJ, Stewart SH, Comeau N, Maclean AM. Efficacy of cognitive-behavioral interventions targeting personality risk factors for youth alcohol misuse. *J Clin Child Adolesc Psychol*. 2006. doi:10.1207/s15374424jccp3504_6

44. Hayes R, Moulton L. Introduction. Cluster Randomised Trials, Interdisciplinary Statistics. 2009.

45. Newman JP. Passive Avoidance in Syndromes of Disinhibition. Psychopathy and Extraversion Measurement of Psychopathy View project. *Artic J Personal Soc Psychol*. 1985. doi:10.1037/0022-3514.48.5.1316

46. Cragg L, Nation K. Self-ordered pointing as a test of working memory in typically developing children. *Memory*. 2007. doi:10.1080/09658210701390750

47. Antonio DW-S, Corporation TTP, 2003 undefined. Wechsler intelligence scale for children–Fourth Edition (WISC-IV).

48. Castellanos-Ryan N, Pihl RO, Conrod PJ. Title: Mechanisms of personality-targeted intervention effects on adolescent alcohol misuse, internalising and externalising symptoms Author list (listed in order of contribution) and contact information. doi:10.1037/ccp0000082

49. Moyers TB, Martin T, Manuel JK, Miller WR, Ernst & D. *Revised Global Scales: Motivational Interviewing Treatment Integrity 3.0 (MITI 3.0)*.

50. Woicik P, Stewart S, Pihl R, behaviors PC-A, 2009 undefined. The substance use risk profile scale: A scale measuring traits linked to reinforcement-specific substance use profiles. *Elsevier*. https://www.sciencedirect.com/science/article/pii/S0306460309001713. Accessed July 30, 2019.

51. Campbell M, Piaggio G, Elbourne D, Bmj DA-, 2012 undefined. Consort 2010 statement: extension to cluster randomised trials. *bmj.com*. https://www.bmj.com/content/345/bmj.e5661. Accessed July 30, 2019.

52. econometrica HW-, 1980 undefined. A heteroskedasticity-consistent covariance matrix estimator and a direct test for heteroskedasticity. *researchgate.net*. https://www.researchgate.net/profile/Alexandre_Janot/publication/268801533_White_Econometrica1980/links/5475dd170cf2778985af1a51.pdf. Accessed July 30, 2019.

53. Lee VE. Using hierarchical linear modeling to study social contexts: The case of school effects. *Educ Psychol*. 2000. doi:10.1207/S15326985EP3502_6

54. Robins JM, Rotnitzky A, Zhao LP. Estimation of regression coefficients when some regressors are not always observed. *J Am Stat Assoc*. 1994. doi:10.1080/01621459.1994.10476818

55. Fiero MH, Huang S, Oren E, Bell ML. Statistical analysis and handling of missing data in cluster randomized trials: A systematic review. *Trials*. 2016. doi:10.1186/s13063-016-1201-z

56. Lewin A, Brondeel R, Benmarhnia T, … FT-, 2018 undefined. Attrition bias related to missing outcome data. *ingentaconnect.com*. https://www.ingentaconnect.com/content/wk/ede/2018/00000029/00000001/art00018. Accessed July 31, 2019.

57. White IR, Royston P, Wood AM. Multiple imputation using chained equations: Issues and guidance for practice. *Stat Med*. 2011. doi:10.1002/sim.4067

58. Sloboda Z, Stephens R, Stephens P, … SG-D and alcohol, 2009 undefined. The Adolescent Substance Abuse Prevention Study: A randomized field trial of a universal substance abuse prevention program. *Elsevier*. https://www.sciencedirect.com/science/article/pii/S0376871609000556. Accessed July 31, 2019.

59. Werch CE, Owen DM. Iatrogenic effects of alcohol and drug prevention programs. *J Stud Alcohol*. 2002. doi:10.15288/jsa.2002.63.581

60. Wilson DB, Gottfredson DC, Najaka SS. School-Based Prevention of Problem Behaviors: A Meta-Analysis. *J Quant Criminol*. 2001. doi:10.1023/A:1011050217296

61. Tobler NS, Roona MR, Ochshorn P, Marshall DG, Streke A V., Stackpole KM. School-based adolescent drug prevention programs: 1998 Meta-analysis. *J Prim Prev*. 2000. doi:10.1023/A:1021314704811

62. Conrod PJ, Castellanos N, Mackie C. Personality-targeted interventions delay the growth of adolescent drinking and binge drinking. *J Child Psychol Psychiatry Allied Discip*. 2008. doi:10.1111/j.1469-7610.2007.01826.x

63. Conrod PJ, Castellanos-Ryan N, Strang J. *Brief, Personality-Targeted Coping Skills Interventions and Survival as a Non-Drug User Over a 2-Year Period During Adolescence*. Vol 67.; 2010.

64. Conrod, P. J., Castellanos-Ryan, N., & Mackie C. Long-term, motivation specific and population-level preventative effects ef personality-targeted interventions for adolescent substance misuse. "Lost in Translation" Conference, Vancouver, February. 2011:and submitted manuscript.

65. Castellanos N, Conrod P. Brief interventions targeting personality risk factors for adolescent substance misuse reduce depression, panic and risk-taking behaviours. *J Ment Heal*. 2006. doi:10.1080/09638230600998912

66. Germain M., G. L., Landry M., Tremblay J., Brunelle N. et Bergeron J. Grille de dépistage de consommation problématique d'alcool et de drogues chez les adolescents et les adolescentes - DEP-ADO. Version 3.1. *Montréal Rech Interv sur les Subst psychoactives - Québec*. 2005.

67. Thush C, Wiers R, Ames S, dependence JG-… alcohol, 2008 undefined. Interactions between implicit and explicit cognition and working memory capacity in the prediction of alcohol use in at-risk adolescents. *Elsevier*. https://www.sciencedirect.com/science/article/pii/S0376871607004322. Accessed August 19, 2019.

68. Institute for Personality and Ability Testing. Measuring intelligence with the Culture Fair tests. Champaign, IL: Institute for Personality and Ability Testing; 1973.

69. Séguin J, Nagin D, … JA-J of A, 2004 undefined. Cognitive-neuropsychological function in chronic physical aggression and hyperactivity. *psycnet.apa.org*. https://psycnet.apa.org/record/2004-20178-011. Accessed August 19, 2019.

70. Cohen M. J. Manual for the children's memory scale. San Antonio, TX: The Psychological Corporation; 1997.

71. Newman J, Review JW-CP, 1993 undefined. Diverse pathways to deficient self-regulation: Implications for disinhibitory psychopathology in children. *Elsevier*. https://www.sciencedirect.com/science/article/pii/S0272735805800029. Accessed August 19, 2019.

72. Cragg L., Nation K. Self-ordered pointing as a test of working memory in typically developing children. Memory 2007; 15: 526–35.

73. Jakobsen JC , Gluud C, Wetterslev J, Winkel P. When and how should multiple imputation be used for handling missing data in randomised clinical trials–a practical guide with flowcharts. BMC Medical Research Methodology. 2017. doi:10.1186/s12874-017-0442-1

31.b Reference to Data Management Plan

Study was conducted under the Good Clinical Data Management Practices (GCDMP) and the Good Clinical Practices (GCP).

31.c Reference to Trial master file and statistical master file

31.d Reference to other Standardized operating procedures or documents

APPENDIX

Appendix 1: Timing of outcome assessments

Primary Outcome

Measure	Time Frame
DEP-ADO: Self-reported alcohol and drug problems (>=18)	At follow-up 4

Secondary Intermediate Outcomes

Measure	Time Frame
Global cognitive function:	
Adaptation of the Cultures Figure Task (CFT) [68]	At each time point
Adaptation of the 'Dot Location' test, a subtest of the Child Memory Scales (CMS)[74]	At each time point
Executive function:	
Adaptation of the Go/No-Go Passive Avoidance Learning Paradigm (PALP) [45,71]	At each time point
'Find the phone' is a task based on the Self-Order Pointing Task[72]	At each time point

Other secondary outcomes

Measure	Time Frame
DEP-AD0[66] age of drinking and substance use onset, binge drinking items	At each time point
SDQ[67]: Conduct problems, peer problems, emotional problems and hyperactivity/inattentive symptoms	At each time point
Depression and anxiety symptom severity (BSI)	At each time point
Unauthorized absences	At each time point
Attendance, absence and drop-out	At each time point

Appendix 2: CONSORT diagram for the final study sample (Co-venture flow diagram)

Figure 2. The percentages in this diagram represent the proportion of participants followed each year on the initial group size at baseline.

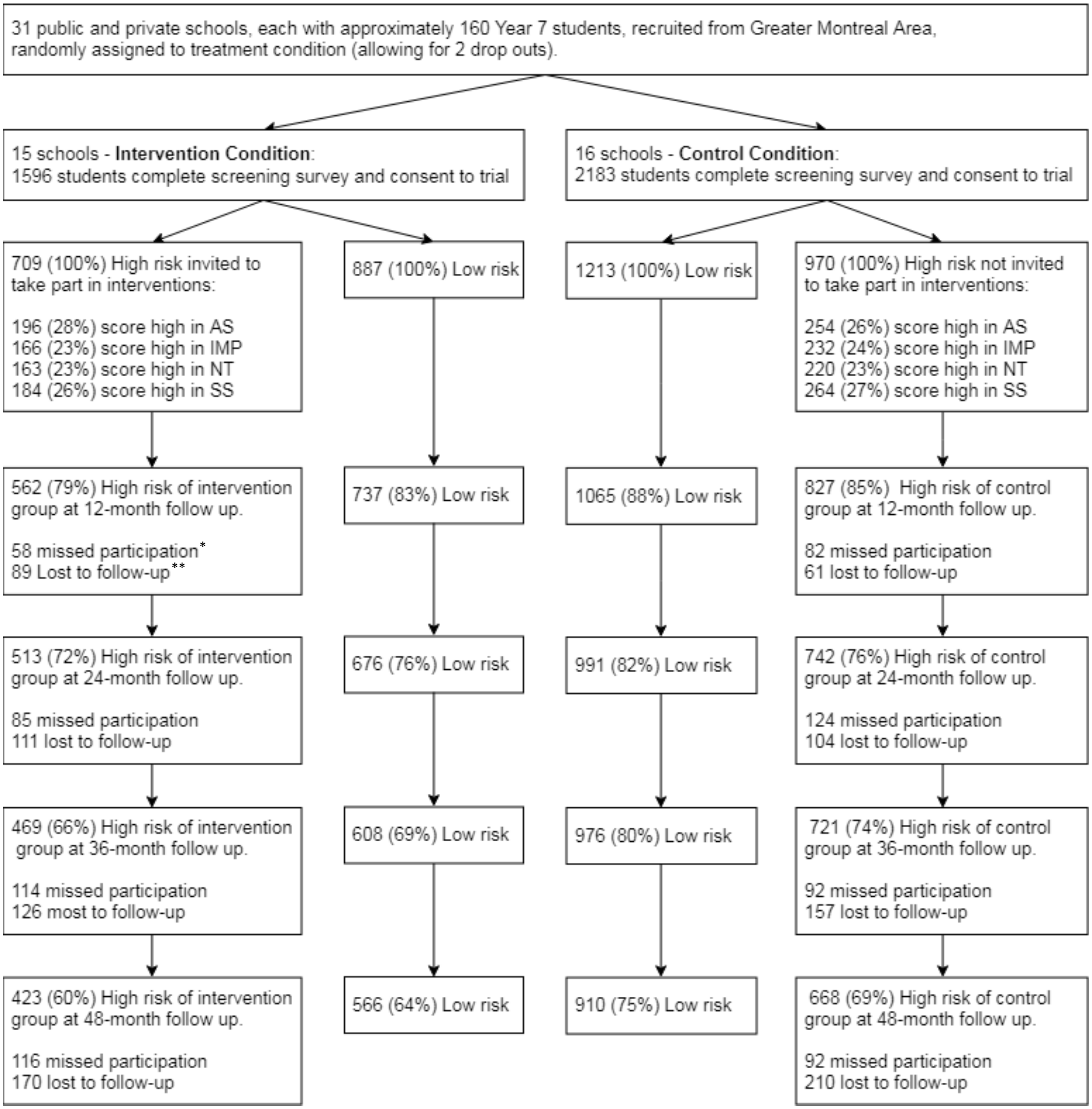

*Missed participation: The participant was absent or refused to participate that specific year.

**Lost to follow-up: The participant has left the school either for academic, conduct, moving reasons.

Appendix 3: Additional information on Outcome measures.

Table 3: Summary of outcomes, measures and time of assessment.

Outcomes	Variable	Measure (items)	Grade at time of assessment
**Primary**	Substance abuse/dependence	DEP-ADO total score >=18 (DEPAPO_ECHELLE_TOTAL)	8, 9, 10, 11
**Secondary**	Global cognitive function	Global cognitive function:	
	• Non-verbal functioning and abstract reasoning	• Adaptation of the CFT (IQ)	8, 9, 10, 11
	• Verbal and visual memory (immediate and delayed recall)	• Adaptation of the CMS (DLR)	8, 9, 10, 11
	Executive functioning	Executive function:	
	• Cognitive control and response inhibition	• Adaptation of the PALP (INH)	8, 9, 10, 11
	• Spatial working memory	• 'Find the phone' task (WM)	8, 9, 10, 11
	Substance use onset and binge	• Substance use onset (DEPAPO_ALC_OC2, DEPAPO_DRUG_OC1)<br>• Binge items (DEPAPO_ALC_OC5, DEPAPO_ALC_OC8)	8, 9, 10, 11
	Mental health symptoms	• Emotional problems scale (SDQ_echelle_emotion_prob)<br>• Conduct problems scale (SDQ_echelle_conduct_prob)<br>• Hyperactivity scale (SDQ_echelle_hyper_prob)<br>• Peer problems scale (SDQ_echelle_peer_prob)<br>• SDQ total score (SDQ_echelle_total_difficulties)	8, 9, 10, 11
		• Depression (BSI_echelle_TotalDepression)<br>• Anxiety (BSI_echelle_TotalAnxiete)	8, 9, 10, 11
	School attendance and drop-out	Unauthorized absences (DEM_06_Y1)<br>Attendance, absence and drop-out (ATT)	8, 9, 10, 11

Description of protocol deviation for the trial

In the end, some schools did require active parental consent for youth to participate in this trial; therefore, not all HR youth were recruited and were provided with interventions in the intervention condition. However, these schools will remain in the study, and considered “compliant”. Only schools that agreed and were able to facilitate follow-up every year were included in the study, regardless of follow-up rate (therefore, compliant). Schools that did not agree to continue to facilitate follow-up of students were considered dropout (non-compliant) and will not be included in the intent to treat analysis. Protocol deviation are defined as departure from the procedures outlined in the protocol. We used a modified intention to treat (ITT) design including only schools that were randomized and followed during 5 years. At the individual level, ITT will be all high-risk students in the schools who consented to a trial and eligible to receive intervention (Regardless of whether or not they received it).

Protocol deviation (PD) was classified prior to unblinding of intervention groups.

1. During the recruitment phase, five schools were recruited and dropped out after the randomisation. Among those, three schools never launched screening and baseline survey, and so were not included. One school dropped out after screening and one school did deliver intervention, but failed to comply with follow-up procedures, so was also dropped from the trial and not included in ITT.
2. Truancy is self-reported and academic records were not obtained.
3. The CMS had to be modified to address ceiling effects in later years of follow-up (one additional item was added in year 3, 4 and 5).
4. School and participant recruitment was completed outside of the planned window since there were two waves of recruitment, students were recruited in the 7th grade in 2012 and2013.
5. Some procedures not performed. We changed intervention adherence criteria: Overall86% of HR students received intervention, but some schools had poor implementation which fell below our initial criteria for evaluation. We will control for adherence, rather than require a certain level of implementation. One school closed, but the school remained in the study, because they provided access to student information so that they could be followed individually. One school missed a follow-up survey in year 4 (due to natural disaster, flood) and instead completed two surveys 7 months apart in the final year: they are considered compliant and in the ITT.
6. **Deviation from planned statistical analyses**: when analysing this dataset, it became apparent that the Proc GLIMMIX method in SAS that is described in the analysis plan was not the most appropriate method to analyse this dataset, as this method required that an imputed dataset be generated in order to include all available data in the analysis. Due to the complex structure of this dataset, and the fact that data were missing at the individual and school level, multiple imputation as a method to manage missing data failed to converge, due to the fact that these models have difficulty managing high-dimensional models with correlated parameters. They either take very long to converge, or don’t converge at all, especially for dichotomous outcomes. Very recently, the benefits of the Bayesian framework for managing multi-level data with missingness have been highlighted as potential solutions for social and health sciences. A number of solutions now exist using the STAN package in R. The MLM in Bayesian framework is especially useful when dealing with repeated measurements (e.g., when measurements are nested within participants) or when handling complex dependency structures in the data such as unequal sample sizes

or missing data. Many statistical packages are developed to fit MLMs but their functionality is limited to the mean of the response distribution, with other parameters of the response distribution, such as the residual standard deviation in linear models, assumed constant across observations. However, when sample sizes or variances change over time, or when missing data occur at different levels in the structure of a dataset, a maximal varying effect structure is needed and many approaches will give aberrant estimations of the correlation between varying effects. While the Proc GLIMMIX provides estimators to correct for this instability, it is generally agreed that Bayesian approaches are better suited to solve this type of problem, especially for dichotomous outcomes with sparse distribution (few non-zeros). It is also preferred to not have to impute datasets in order to replace missing data, which the Bayesian approach allows. Therefore, a slight deviation from the original analysis strategy involved moving from a frequentist to a BayesianMLM model. STAN uses the brms package and supports diverse distributions and link functions, allowing for multiple grouping factors each with multiple group-level effects. It also allows for autocorrelation of the response variable, user defined covariance structures, an flexibleand explicit prior specifications.

The script used to create the primary outcome analysis is as follows:

```
##### model estimating linear growth over time #####

model_depado <- brm(Flagged ~ 1 + DEM_01 + Exp_Group + Language + year
+year:Exp_Group + (1|id+schoolnum),
data = cov_5,
family = bernoulli,
warmup = 1000,
iter = 3000,
cores = 4,
chains = 4,
seed = 123)
sum_model_depado<-summary(model_depado)

##### model with categorical time variable #####

cov_5$year_cat=as.factor(cov_5$year)

model_depado_cat <- brm(Flagged ~ 1 + DEM_01 + Exp_Group + Language +year_cat +
year_cat:Exp_Group + (1|id+schoolnum),
data = cov_5,
family = bernoulli,
warmup = 1000,
iter = 3000,
cores = 4,
chains = 4,
seed = 123)
sum_model_depado_cat<-summary(model_depado_cat)
```
