# Supplementary material for: Selective Personality-Targeted Intervention and the Escalation of Substance Use During Adolescence: A Secondary Analysis of A Cluster-Randomized Clinical Trial
Source: JAMA Netw Open. 2025 Dec 18;8(12):e2550176. doi: 10.1001/jamanetworkopen.2025.50176 (PMC12715647; doi:10.1001/jamanetworkopen.2025.50176)
Supplement: Supplement 2. — eTable 1. Substance use outcomes at all measurement occasions eTable 2. Baseline differences between those who missed any follow ups and those present for all follow ups adjusting for school-level variance using multilevel logistic regression models eTable 3. Baseline differences between those who missed all follow ups and those present for at least one follow up adjusting for school-level variance using multilevel logistic regression models eTable 4. Model comparison of substance use trajectories: LOO-CV fit indices with linear and quadratic effects eTable 5. Bayesian estimates, standard errors, credible intervals and odds ratios for the effect of the intervention on the count of illicit substances used [file jamanetwopen-e2550176-s002.pdf]

Supplemental Online Content

Lynch SJ, Stewart SH, Conrod P. Selective personality-targeted intervention and escalation of substance use during adolescence: a secondary analysis of a cluster-randomized clinical trial. *JAMA Netw Open*. 2025;8(12):e2550176.
doi:10.1001/jamanetworkopen.2025.50176

eTable 1. Substance use outcomes at all measurement occasions
eTable 2. Baseline differences between those who missed any follow ups and those present for all follow ups adjusting for school-level variance using multilevel logistic regression models
eTable 3. Baseline differences between those who missed all follow ups and those present for at least one follow up adjusting for school-level variance using multilevel logistic regression models
eTable 4. Model comparison of substance use trajectories: LOO-CV fit indices with linear and quadratic effects
eTable 5. Bayesian estimates, standard errors, credible intervals and odds ratios for the effect of the intervention on the count of illicit substances used

This supplemental material has been provided by the authors to give readers additional information about their work.

© 2025 Lynch SJ et al. *JAMA Network Open*.

Software

Analyses were conducted in R (version 4.4.1; R Core Team, 2024) on macOS 15.3.1, using the brms (version 2.22.0; Bürkner P, 2017) and loo (version 2.8.0; Vehtari A et al., 2024) packages.

Substance use outcomes at all measurement occasions

Table S1. Substance use outcomes at all measurement occasions

	Year 1 (Baseline)		Year 2		Year 3		Year 4		Year 5	
	Control	Intervention	Control	Intervention	Control	Intervention	Control	Intervention	Control	Intervention
**Alcohol**										
Never	558 / 964 (58%)	392 / 705 (56%)	365 / 818 (45%)	238 / 554 (43%)	262 / 733 (36%)	166 / 504 (33%)	174 / 708 (25%)	115 / 465 (25%)	125 / 658 (19%)	82 / 421 (19%)
Occasionally	335 / 964 (35%)	259 / 705 (37%)	350 / 818 (43%)	242 / 554 (44%)	312 / 733 (43%)	244 / 504 (48%)	283 / 708 (40%)	218 / 465 (47%)	267 / 658 (41%)	181 / 421 (43%)
Approx. Once a month	44 / 964 (4.6%)	35 / 705 (5.0%)	75 / 818 (9.2%)	55 / 554 (9.9%)	97 / 733 (13%)	63 / 504 (13%)	135 / 708 (19%)	75 / 465 (16%)	125 / 658 (19%)	80 / 421 (19%)
1-2 times / week OR weekends	21 / 964 (2.2%)	16 / 705 (2.3%)	25 / 818 (3.1%)	17 / 554 (3.1%)	60 / 733 (8.2%)	25 / 504 (5.0%)	103 / 708 (15%)	51 / 465 (11%)	132 / 658 (20%)	70 / 421 (17%)
3+ times / week	4 / 964 (0.4%)	0 / 705 (0%)	3 / 818 (0.4%)	2 / 554 (0.4%)	2 / 733 (0.3%)	4 / 504 (0.8%)	10 / 708 (1.4%)	2 / 465 (0.4%)	7 / 658 (1.1%)	7 / 421 (1.7%)
Everyday	2 / 964 (0.2%)	3 / 705 (0.4%)	0 / 818 (0%)	0 / 554 (0%)	0 / 733 (0%)	2 / 504 (0.4%)	3 / 708 (0.4%)	4 / 465 (0.9%)	2 / 658 (0.3%)	1 / 421 (0.2%)
(Missing)			146	151	231	201	256	240	306	284
**Cannabis**										
Never	909 / 964 (94%)	641 / 705 (91%)	722 / 818 (88%)	470 / 554 (85%)	554 / 733 (76%)	368 / 504 (73%)	456 / 708 (64%)	301 / 465 (65%)	373 / 658 (57%)	254 / 421 (60%)
Occasionally	33 / 964 (3.4%)	33 / 705 (4.7%)	60 / 818 (7.3%)	53 / 554 (9.6%)	107 / 733 (15%)	78 / 504 (15%)	143 / 708 (20%)	101 / 465 (22%)	143 / 658 (22%)	88 / 421 (21%)
Approx. Once a month	8 / 964 (0.8%)	11 / 705 (1.6%)	15 / 818 (1.8%)	15 / 554 (2.7%)	16 / 733 (2.2%)	17 / 504 (3.4%)	30 / 708 (4.2%)	21 / 465 (4.5%)	43 / 658 (6.5%)	31 / 421 (7.4%)
1-2 times / week OR weekends	4 / 964 (0.4%)	9 / 705 (1.3%)	15 / 818 (1.8%)	10 / 554 (1.8%)	40 / 733 (5.5%)	24 / 504 (4.8%)	38 / 708 (5.4%)	15 / 465 (3.2%)	45 / 658 (6.8%)	22 / 421 (5.2%)
3+ times / week	3 / 964 (0.3%)	8 / 705 (1.1%)	3 / 818 (0.4%)	4 / 554 (0.7%)	5 / 733 (0.7%)	10 / 504 (2.0%)	15 / 708 (2.1%)	14 / 465 (3.0%)	19 / 658 (2.9%)	19 / 421 (4.5%)
Everyday	7 / 964 (0.7%)	3 / 705 (0.4%)	3 / 818 (0.4%)	2 / 554 (0.4%)	11 / 733 (1.5%)	7 / 504 (1.4%)	26 / 708 (3.7%)	13 / 465 (2.8%)	35 / 658 (5.3%)	7 / 421 (1.7%)
(Missing)			146	151	231	201	256	240	306	284
**Smoking (Past 12 months)**										
Never	22 / 70 (31%)	15 / 63 (24%)	19 / 94 (20%)	11 / 77 (14%)	27 / 142 (19%)	16 / 101 (16%)	27 / 180 (15%)	28 / 113 (25%)	36 / 202 (18%)	25 / 115 (22%)
Occasionally	32 / 70 (46%)	29 / 63 (46%)	49 / 94 (52%)	42 / 77 (55%)	69 / 142 (49%)	53 / 101 (52%)	90 / 180 (50%)	46 / 113 (41%)	99 / 202 (49%)	59 / 115 (51%)
Approx. Once a month	9 / 70 (13%)	4 / 63 (6.3%)	12 / 94 (13%)	6 / 77 (7.8%)	16 / 142 (11%)	13 / 101 (13%)	12 / 180 (6.7%)	7 / 113 (6.2%)	14 / 202 (6.9%)	5 / 115 (4.3%)
1-2 times / week OR weekends	1 / 70 (1.4%)	3 / 63 (4.8%)	8 / 94 (8.5%)	4 / 77 (5.2%)	11 / 142 (7.7%)	10 / 101 (9.9%)	18 / 180 (10%)	10 / 113 (8.8%)	17 / 202 (8.4%)	8 / 115 (7.0%)
3+ times / week	0 / 70 (0%)	4 / 63 (6.3%)	2 / 94 (2.1%)	4 / 77 (5.2%)	12 / 142 (8.5%)	3 / 101 (3.0%)	11 / 180 (6.1%)	8 / 113 (7.1%)	17 / 202 (8.4%)	5 / 115 (4.3%)
Everyday	6 / 70 (8.6%)	8 / 63 (13%)	4 / 94 (4.3%)	10 / 77 (13%)	7 / 142 (4.9%)	6 / 101 (5.9%)	22 / 180 (12%)	14 / 113 (12%)	19 / 202 (9.4%)	13 / 115 (11%)
(Missing)	894	642	870	628	822	604	784	592	762	590
**Opioids**										
Never	961 / 964 (100%)	701 / 705 (99%)	805 / 818 (98%)	547 / 554 (99%)	718 / 733 (98%)	490 / 504 (97%)	675 / 708 (95%)	446 / 465 (96%)	628 / 658 (95%)	403 / 421 (96%)
Occasionally	2 / 964 (0.2%)	2 / 705 (0.3%)	11 / 818 (1.3%)	6 / 554 (1.1%)	12 / 733 (1.6%)	11 / 504 (2.2%)	27 / 708 (3.8%)	14 / 465 (3.0%)	25 / 658 (3.8%)	16 / 421 (3.8%)
Approx. Once a month	1 / 964 (0.1%)	0 / 705 (0%)	1 / 818 (0.1%)	1 / 554 (0.2%)	3 / 733 (0.4%)	2 / 504 (0.4%)	1 / 708 (0.1%)	1 / 465 (0.2%)	1 / 658 (0.2%)	2 / 421 (0.5%)
1-2 times / week OR weekends	0 / 964 (0%)	0 / 705 (0%)	0 / 818 (0%)	0 / 554 (0%)	0 / 733 (0%)	1 / 504 (0.2%)	2 / 708 (0.3%)	0 / 465 (0%)	2 / 658 (0.3%)	0 / 421 (0%)
3+ times / week	0 / 964 (0%)	0 / 705 (0%)	1 / 818 (0.1%)	0 / 554 (0%)	0 / 733 (0%)	0 / 504 (0%)	0 / 708 (0%)	0 / 465 (0%)	2 / 658 (0.3%)	0 / 421 (0%)
Everyday	0 / 964 (0%)	2 / 705 (0.3%)	0 / 818 (0%)	0 / 554 (0%)	0 / 733 (0%)	0 / 504 (0%)	3 / 708 (0.4%)	4 / 465 (0.9%)	0 / 658 (0%)	0 / 421 (0%)
(Missing)			146	151	231	201	256	240	306	284
**Illicit Polysubstance Use**										
0	963 / 964 (100%)	697 / 705 (99%)	813 / 818 (99%)	552 / 554 (100%)	731 / 733 (100%)	502 / 504 (100%)	701 / 708 (99%)	460 / 465 (99%)	649 / 658 (99%)	418 / 421 (99%)
1	1 / 964 (0.1%)	8 / 705 (1.1%)	5 / 818 (0.6%)	2 / 554 (0.4%)	2 / 733 (0.3%)	2 / 504 (0.4%)	7 / 708 (1.0%)	5 / 465 (1.1%)	9 / 658 (1.4%)	3 / 421 (0.7%)
(Missing)			146	151	231	201	256	240	306	284

© 2025 Lynch SJ et al. *JAMA Network Open*.

Attrition Analysis

Missing any of the follow ups (vs. completing all follow ups) was associated with higher levels of hopelessness. Missing all follow ups (vs. completing any follow up) was associated with higher levels of all four SURPS personality traits. Attrition differed between intervention and control conditions, but this was not significant after adjusting for school-level variance. No other baseline predictors (substance use, anxiety, depression, language, sex) were associated with attrition after adjusting for school-level variance. As school-level variance is accounted for in all analyses, it was not necessary to include additional covariates to analyses.

Table S2. Baseline Differences Between Those Who Missed Any Follow Ups and Those Present for all follow ups adjusting for school-level variance using multilevel logistic regression models

				95% CI	
**Baseline Characteristic**	**Beta**	**SE**	**p**	**Lower**	**Upper**
Cannabis (Frequency)	0.23	0.17	0.18	-0.11	0.56
Cannabis (Any use)	0.20	0.38	0.59	-0.54	0.95
Prescription drugs (Any use)	-0.11	0.32	0.73	-0.72	0.51
Illicit substances (Any use)	0.03	0.24	0.91	-0.44	0.50
BSI Anxiety	-0.03	0.02	0.12	-0.06	0.01
BSI Depression	0.01	0.01	0.44	-0.02	0.04
**SURPS Negative Thinking**	**0.04**	**0.02**	**0.01**	**0.01**	**0.08**
SURPS Impulsivity	0.03	0.02	0.06	0.00	0.07
SURPS Sensation Seeking	0.02	0.02	0.30	-0.01	0.05
SURPS Anxiety Sensitivity	-0.01	0.02	0.63	-0.04	0.03
Sex (Female)	-0.11	0.12	0.34	-0.34	0.12
Language (French)	-0.37	0.22	0.09	-0.81	0.06
Trial Group (Intervention)	0.15	0.22	0.48	-0.27	0.58

Table S3. Baseline Differences Between Those Who Missed All Follow Ups and Those Present for at least one follow up adjusting for school-level variance using multilevel logistic regression models

				95% CI	
**Baseline Characteristic**	**Beta**	**SE**	**p**	**Lower**	**Upper**
Cannabis (Frequency)	0.12	0.17	0.49	-0.22	0.46
Cannabis (Any use)	0.56	0.45	0.22	-0.33	1.45
Prescription drugs (Any use)	-0.26	0.50	0.60	-1.23	0.71
Illicit substances (Any use)	0.06	0.39	0.88	-0.70	0.82
BSI Anxiety	-0.04	0.03	0.19	-0.10	0.02
BSI Depression	0.00	0.02	0.91	-0.04	0.05
**SURPS Negative Thinking**	**0.08**	**0.03**	**0.00**	**0.03**	**0.13**
**SURPS Impulsivity**	**0.07**	**0.03**	**0.04**	**0.00**	**0.13**
**SURPS Sensation Seeking**	**0.07**	**0.03**	**0.01**	**0.02**	**0.12**

© 2025 Lynch SJ et al. *JAMA Network Open*.

**SURPS Anxiety Sensitivity**	**0.06**	**0.03**	**0.07**	**0.00**	**0.11**
Sex (Female)	0.04	0.20	0.84	-0.34	0.42
Language (French)	0.21	0.34	0.53	-0.45	0.87
Trial Group (Intervention)	0.40	0.33	0.22	-0.24	1.04

© 2025 Lynch SJ et al. *JAMA Network Open*.

Linear vs Quadratic Growth: leave-one-out cross-validation (LOO-CV)

Linear and quadratic growth were compared based on leave-one-out cross-validation (LOO), with lower expected log predictive density (ELPD) indicating better predictive accuracy. Where there was evidence of quadratic growth, additional analyses comparing models with and without a Year^2 x group interaction term were conducting. Results from these comparisons are presented below in Table S4.

Table S4. Model Comparison of Substance Use Trajectories: LOO-CV Fit Indices with Linear and Quadratic Effects

Model	$ELPD_{diff}$	SE $ELPD_{diff}$	ELPD LOO	SE ELPD LOO	p LOO	SE p LOO	LOOIC	SE LOOIC
**Frequency Outcomes**								
**Alcohol (Linear)**	**0.000**	**0.000**	**-5875.270**	**69.482**	**1222.401**	**20.070**	**11750.530**	**138.964**
Alcohol (Quadratic, with Year^2 x group interaction)	-0.322	2.464	-5875.590	69.520	1225.236	20.131	11751.180	139.040
Cannabis (Quadratic, with Year^2 x group interaction)	0.000	0.000	-3655.710	78.205	926.448	26.374	7311.418	156.411
Cannabis (Linear)	-24.345	7.665	-3680.050	77.681	920.795	25.593	7360.108	155.363
**Cannabis (Quadratic, no Year^2 x group interaction)**	0.000	0.000	-3653.860	78.197	924.409	26.304	7307.719	156.394
Cannabis (Quadratic, with Year^2 x group interaction)	-1.850	2.458	-3655.710	78.205	926.448	26.374	7311.418	156.411
Smoking Past 12m (Quadratic, with Year^2 x group interaction)	0.000	0.000	-1632.930	28.571	268.670	8.165	3265.865	57.141
Smoking Past 12m (Linear)	-0.506	2.286	-1633.440	28.445	264.885	8.018	3266.876	56.889
**Smoking Past 12m (Quadratic, no Year^2 x group interaction)**	**0.000**	**0.000**	**-1632.270**	**28.526**	**266.428**	**8.073**	**3264.537**	**57.053**
Smoking Past 12m (Quadratic, with Year^2 x group interaction)	-0.664	0.766	-1632.930	28.571	268.670	8.165	3265.865	57.141
Opioids (Quadratic, with Year^2 x group interaction)	0.000	0.000	-709.377	50.018	217.535	17.940	1418.754	100.035
Opioids (Linear)	-1.869	3.171	-711.246	49.842	214.182	17.614	1422.492	99.684
**Opioids (Quadratic, no Year^2 x group interaction)**	0.000	0.000	-707.275	49.808	215.672	17.752	1414.551	99.616
Opioids (Quadratic, with Year^2 x group interaction)	-2.101	0.897	-709.377	50.018	217.535	17.940	1418.754	100.035
**Binary Outcomes**								
**Polysubstance Use (Linear)**	**0.000**	**0.000**	**-257.140**	**32.413**	**87.048**	**12.809**	**514.280**	**64.827**
Polysubstance Use (Quadratic, with Year^2 x group interaction)	-2.013	1.373	-259.153	32.682	89.318	13.101	518.306	65.364

Note. **$ELPD_{diff}$** = Difference in expected log pointwise predictive density (ELPD) between the current model and the best-performing model; **SE $ELPD_{diff}$** = Standard error of the ELPD difference; **ELPD LOO** = Expected log pointwise predictive density from leave-one-out cross-validation (LOO-CV); **SE ELPD LOO** = Standard error of the ELPD from LOO-CV; **p LOO** = Effective number of parameters in the model; **SE p LOO** = Standard error of the effective number of parameters; **LOOIC** = Leave-one-out information criterion; **SE LOOIC** = Standard error of the LOOIC.

© 2025 Lynch SJ et al. *JAMA Network Open*.

Sensitivity Analysis: Illicit Polysubstance Use

Table S5. Bayesian estimates, standard errors, credible intervals and odds ratios for the effect of the intervention on the count of illicit substances used

	B	SE	95% CI (Lower)	95% CI (Upper)
**(Intercept)**	**-3.480**	**0.468**	**-4.429**	**-2.580**
Trial Group (Intervention)	0.443	0.386	-0.326	1.211
Year	-0.070	0.210	-0.473	0.353
Year^2	0.059	0.050	-0.039	0.154
Sex (Female)	-0.391	0.216	-0.807	0.038
Language (French)	-0.190	0.322	-0.827	0.451
**Intervention x Year**	**-0.340**	**0.125**	**-0.584**	**-0.088**

© 2025 Lynch SJ et al. *JAMA Network Open*.
