# Supplementary material for: Selective Personality-Targeted Intervention and the Escalation of Substance Use During Adolescence: A Secondary Analysis of A Cluster-Randomized Clinical Trial
Source: JAMA Netw Open. 2025 Dec 18;8(12):e2550176. doi: 10.1001/jamanetworkopen.2025.50176 (PMC12715647; doi:10.1001/jamanetworkopen.2025.50176)
Supplement: Supplement 3. — Data Sharing Statement [file jamanetwopen-e2550176-s003.pdf]

Data Sharing Statement

Lynch. Selective Personality-Targeted Intervention and Escalation of Substance Use During Adolescence. *JAMA Netw Open*. Published December 18, 2025.
doi:10.1001/jamanetworkopen.2025.50176

Data
Additional Information: ClinicalTrials.gov identifier: NCT01655615
Data available: No

Additional Information
Explanation for why data not available: Data may be made available upon reasonable request.
